# An oropharyngeal gonorrhoea controlled human infection model: a provisional protocol using a novel *Neisseria gonorrhoeae* challenge strain

**DOI:** 10.12688/wellcomeopenres.25103.1

**Published:** 2025-11-19

**Authors:** Eloise Williams, Georgina L Pollock, David J Price, Tim Crocker-Buque, Dana de Kretser, Euzebiusz Jamrozik, Joshua Osowicki, Shivani Pasricha, Francesca Azzato, Andrew Steer, Joanna R Groom, Danika L Hill, Jason A Roberts, Wilhelmina M Huston, Kate L Seib, Christopher K Fairley, Eric PF Chow, Marcus Y Chen, Jane S Hocking, Deborah A Williamson, James S McCarthy

**Affiliations:** 1 Department of Infectious Diseases, The University of Melbourne at the Peter Doherty Institute for Infection and Immunity, Melbourne, Victoria, 3000, Australia; 2 Victorian Infectious Diseases Reference Laboratory, Royal Melbourne Hospital at the Peter Doherty Institute for Infection and Immunity, Melbourne, Victoria, 3000, Australia; 3 Department of Infectious Diseases and Immunology, Austin Health, Melbourne, Victoria, 3000, Australia; 4 The Walter and Eliza Hall Institute of Medical Research, Melbourne, Victoria, 3000, Australia; 5 Centre for Epidemiology and Biostatistics, Melbourne School of Population and Global Health, The University of Melbourne, Melbourne, Victoria, 3000, Australia; 6 Ethox and Pandemic Sciences Institute, University of Oxford, Oxford, England, 38665, UK; 7 Tropical Diseases Research Group, Murdoch Children's Research Institute, Melbourne, Victoria, 3000, Australia; 8 Department of Paediatrics, The University of Melbourne, Melbourne, Victoria, 3000, Australia; 9 Department of Infectious Diseases, Royal Children's Hospital, Royal Children's Hospital, Melbourne, Victoria, 3000, Australia; 10 Department of Medical Biology, The University of Melbourne, Melbourne, Victoria, 3000, Australia; 11 Department of Immunology, Monash University, Monash University, Clayton, Victoria, 3000, Australia; 12 University of Queensland Centre for Clinical Research, University of Queensland, Brisbane, Queensland, 4000, Australia; 13 Herston Infectious Diseases Institute, MetroNorth Health, Brisbane, Queensland, 4000, Australia; 14 Departments of Pharmacy and Intensive Care Medicine, Royal Brisbane and Women's Hospital, Melbourne, Queensland, 4000, Australia; 15 Division of Anesthesia Critical Care and Emergency Pain Medicine, Nimes University Hospital, UR UM 103, University of Montpellier, Nimes, France; 16 Faculty of Science, The University of Melbourne, Melbourne, Victoria, 3000, Australia; 17 Institute for Biomedicine and Glycomics, Griffith University, Gold Coast, Queensland, 4222, Australia; 18 Melbourne Sexual Health Centre, Alfred Health, Melbourne, Victoria, 3000, Australia; 19 School of Translational Medicine, Monash University, Melbourne, Victoria, 3800, Australia; 20 Sexual Health Unit, Melbourne School of Population and Global Health, The University of Melbourne, Melbourne, Victoria, 3000, Australia; 21 School of Medicine, University of St Andrews, Fife, Scotland, UK; 22 Victorian Infectious Diseases Service, The Royal Melbourne Hospital at the Peter Doherty Institute for Infection and Immunity, Melbourne, Victoria, 3000, Australia

**Keywords:** Controlled human infection model, gonorrhoea, sexually transmitted infection, study protocol, vaccine, antimicrobial resistance

## Abstract

**Introduction:**

Gonorrhoea is a sexually transmissable infection caused by *Neisseria gonorrhoeae* that causes a significant global burden of disease. Urogenital infection can result in long-term impacts on reproductive, perinatal, and neonatal health. Little progress has been made in the public health control of gonorrhoea, and novel preventative strategies are urgently needed. Furthermore, future gonorrhoea management is threatened by increasing antimicrobial resistance (AMR). Oropharyngeal gonorrhoea is usually asymptomatic; likely plays an important role in development of AMR; and is a high-risk site for treatment failure. Here, we describe a protocol for an oropharyngeal gonorrhoea controlled human infection model (CHIM) that has been designed to maximize participant safety, with the aim of developing this as a platform to accelerate prevention and treatment strategies.

**Methods and analysis:**

This dose-escalation CHIM study will enrol 20-35 healthy adult volunteers aged 18 to 50 years who were assigned male at birth and only have sex with people assigned male at birth. The primary objectives are to determine i) the safety and tolerability of an oropharyngeal gonorrhoea CHIM; and, ii) the minimum infectious dose of isolate AUSMDU00053933 required for 60–80% of participants to develop oropharyngeal *N. gonorrhoeae* infection. Secondary and exploratory endpoints include description of clinical, immunological, microbiological and pharmacometric responses. Participants will be monitored daily as outpatients during the five-day experimental infection phase. All participants will be treated with antibiotics, and followed up for three months. Statistical analysis and dose escalation/de-escalation decisions will follow a model-based continual reassessment method in a Bayesian statistical framework.

**Ethics and dissemination:**

After scientific peer review of this provisional protocol, a detailed protocol will be submitted for human research ethics committee assessment. Protocol development was informed by feedback from community engagement. Study findings will be disseminated in peer-reviewed journals and at scientific meetings, with summaries provided to relevant stakeholders.

## Plain Language summary

This report outlines a research study protocol using a controlled human infection model, where participants are inoculated in the throat with *Neisseria gonorrhoeae*, the bacteria that causes gonorrhoea. Throat infections usually have no symptoms but can still lead to unintentional spread to others. Genital gonorrhoea can cause painful symptoms. In rare cases, the infection can spread through the bloodstream to affect joints, tendons, and skin. It can also cause serious long-term health problems in women, including infertility. Although gonorrhoea can be treated with antibiotics, the only effective treatment is ceftriaxone and ceftriaxone-resistant gonorrhoea is increasingly reported. This increasing resistance makes it vital to develop new treatments. This initial study aims to confirm that the research approach is safe and to find the dose of bacteria that reliably causes throat infection within five days. All participants will be treated with antibiotics within five days of being exposed to the bacteria. As gonorrhoea can cause long-term reproductive impacts in people with a female reproductive system, only adults aged 18 to 50 who were assigned male at birth and have sex exclusively with others assigned male at birth will be eligible. Participants will attend the clinic as outpatients and must agree not to have any form of sexual contact, including oral sex and kissing, until they are cured. The study will explore clinical symptoms, immune responses, and how the bacteria behave in the throat. This will improve understanding of throat gonorrhoea. If this early research is successful, the model will be used to test new antibiotics, vaccines, and prevention strategies, with the ultimate goal of reducing the global burden of disease due to gonorrhoea.

## Introduction

*Neisseria gonorrhoeae* is a significant global pathogen ^1^, estimated to infect approximately 88 million people per year ^2^. The greatest burden of disease is carried by people in low- and middle-income countries where there has been no improvement in rates of infection in the past 30 years ^2^. In high-income settings, there has been a dramatic increase in rates of infection over the past decade, with infection most prevalent in high-risk populations including men who have sex with men (MSM), transgender persons, sex workers and socially marginalised populations including indigenous populations ^1^. *N. gonorrhoeae* can cause symptomatic urogenital gonorrhoea ^3^, sight-threatening gonococcal conjunctivitis ^4^, and uncommonly, disseminated gonococcal infection ^5^. Left untreated, urogenital infection can result in adverse reproductive outcomes including pelvic inflammatory disease, infertility, and complications in pregnancy ^6^. Infection therefore disproportionately impacts women and their children. Urogenital infection may also increase the risk of HIV acquisition ^7^. *N. gonorrhoeae* also causes infection at extragenital sites including the oropharynx and anorectum; however, infection at these anatomical sites is usually asymptomatic ^8^.

Asymptomatic infection, particularly of the oropharynx, may play an important role in gonorrhoea transmission ^9^. Antimicrobial resistance (AMR) in *N. gonorrhoeae* has been designated a critical threat to public health by the World Health Organization and by the United States Centers for Disease Control and Prevention ^10, 11^. *N.gonorrhoeae* exhibits a remarkable capacity to develop antimicrobial resistance (AMR), driven by its high genetic plasticity and ability to readily acquire resistance determinants through horizontal gene transfer ^12^. Oropharyngeal infection is a high-risk site for acquisition of AMR, due to prolonged colonisation ^13^ and potential for horizontal gene transfer from commensal *Neisseria* species and other organisms ^12^.

Controlled human infection models (CHIMs) have contributed to the development of new vaccines and therapeutics for infectious diseases of concern such as typhoid, malaria, and influenza ^14^. A male gonorrhoea urethritis CHIM, established in the United States in the 1980s ^15^, has predominantly been used to study pathogenesis. More recently, it has been used to evaluate vaccine efficacy ^16^. An oropharyngeal gonorrhoea CHIM may be more acceptable to potential participants due to the lower likelihood of symptomatic infection and relatively non-invasive inoculation procedure compared to the urethritis model. An oropharyngeal gonorrhoea CHIM may play a key role in i) accelerating gonorrhoea vaccine development; ii) assessing the efficacy of novel antimicrobials at the important oropharyngeal site of infection; iii) identifying an immune correlate of protection to support future vaccine development and optimization; and, iv) characterising *N. gonorrhoeae* infection dynamics in the oropharynx ^17^.

Following multiple observational studies suggesting partial efficacy of an outer membrane vesicle (OMV) *Neisseria meningitidis* serogroup B vaccine (4CMenB) against gonorrhoea ^18^, and the increasingly urgent need for new preventative strategies, there is renewed interest in development of a gonococcal vaccine. However, results from the first randomised controlled trial of 4CMenB in MSM failed to demonstrate efficacy against gonorrhoea infection ^19^. Despite these discouraging results, there remains hope for the development of an effective gonorrhoea vaccine. There are several gonorrhoea vaccines in early clinical and late preclinical development using OMV technology optimized specifically for *N. gonorrhoeae*, as well as vaccines incorporating novel vaccine antigens and adjuvants ^20^. Novel antimicrobials have also recently been developed against *N. gonorrhoeae*, including zoliflodacin ^21^ and gepotidicin ^22^, with phase three studies demonstrating that these first-in-class antimicrobials are non-inferior to standard care for treatment of uncomplicated urogenital infection. However, these agents may have sub-maximal efficacy against oropharyngeal infection, the most challenging site for antimicrobial efficacy against gonorrhoea ^23^. Unless new antimicrobials, vaccines, and other strategies have efficacy in the oropharynx, there is a risk that they will fail to control gonorrhoea at a population level ^23, 24^. 

Here, we describe the study design considerations, and a protocol for a first-in-human oropharyngeal gonorrhoea CHIM. Ethical considerations, including the scientific justification, safety and risk mitigation have been carefully considered and are described elsewhere ^17^. The primary consideration in the development of this protocol has been to maximize participant and community safety. Development of this trial protocol has also followed best practice by incorporating community consultation into the study design phase ^25, 26^. This community engagement process has shaped protocol development by informing recruitment strategies, informed consent materials, and study procedures and will be described in a forthcoming publication. Other critical steps towards establishing a safe, and informative model have been: i) the genomics-based selection of a novel challenge strain from a large contemporary collection of clinical *N. gonorrhoeae* isolates ^27^; and, ii) developing an animal-free liquid media strain manufacture that enables preparation of single-use frozen dose vials at various concentrations that can be thawed for use on the day of inoculation and in-depth release testing prior to participant inoculation, which will be published separately.

## Study design considerations

The success of our first-in-human CHIM requires a clear rationale for study procedures and endpoints. Lessons may be drawn from the experience of several other CHIMs ( Table 1). Critical to the design of an oropharyngeal gonorrhoea CHIM is building upon the decades of experience with the male urethritis model, which was established over 40 years ago ^15^. However, given oropharyngeal gonorrhoea is predominantly an asymptomatic infection, many study design considerations are comparable with other human models of bacterial upper respiratory tract colonisation, such as *Neisseria lactamica* ^28^, *Bordetella pertussis* ^29^, and *Streptococcus pneumoniae* ^30^. Much can also be learnt from the *Streptococcus pyogenes* model ^31^, the only other CHIM including an oropharyngeal challenge. Gastrointestinal bacterial CHIMs such as *Salmonella enterica* serovar Typhi ^36^, *Salmonella enterica* serovar Paratyphi A ^37^, and non-typhoidal *Salmonella* ^39^ are also informative in that each involve infection with a transmissible bacterial pathogen with a large and highly diverse genome, similar to *N. gonorrhoeae*.

**Table 1.** Key design features of bacterial controlled human infection models relevant to the design of an oropharyngeal gonorrhoea model.

Organism	Participant eligibility	Inoculation (route, dose, attack rate achieved)	Setting	Duration between inoculation and treatment (or end of monitoring if treatment not provided)	Treatment (anti-microbial, dose, duration and timing)	Key features applicable to oropharyngeal gonorrhoea CHIM	Reference
Colonization studies							
*Bordetella pertussis*	Healthy adults aged 18–45 years without known or suspected recent pertussis infection	Nasal, ~10 ^5^ cfu, 80% attack rate	Inpatient	14 days	Oral azithromycin 500mg daily for 3 days at day 14	Infectious upper respiratory pathogen, treatment at end of observation period	De Graaf *et al.*, Clin Infect Dis, 2020 ^32^
*Streptococcus pneumoniae*	Healthy adults aged 18–50 years	Nasal, ~10 ^5^ cfu, 70% attack rate	Outpatient	14 days	Oral amoxicillin 500mg tds for 3 days at day 14	Infectious upper respiratory pathogen, treatment at end of observation period, outpatient design	Robinson *et al.*, Am J Respir Crit Car, 2022 ^33^
*Neisseria lactamica*	Healthy adults aged 18–45 years without active *N. lactamica* carriage or meningococcal vaccination in prior 5 years	Nasal, ~10 ^4^ cfu, 60% attack rate	Outpatient	24 weeks	Nil	*Neisseria* species upper respiratory tract inoculation, 10 ^4^ cfu dose with 60% attack rate. Subsequent CHIM involving genetically modified *N. lactamica* involving recruitment of contact participants as well as study participants	Evans, Clin Infect Dis, 2011 ^34^ Gbesemete *et al.*, BMJ Open, 2019 ^35^
Clinical endpoint studies							
*Neisseria gonorrhoeae*	Healthy adults assigned male sex at birth, aged 18–35 years	Urethral, ~10 ^3^ (MS11mkC), 50% attack rate; ~10 ^6^ (FA1090), 80–90% attack rate	Inpatient-leave unit during day; Outpatient	5–10 days	Oral cefixime 400mg single dose at symptoms or day 5	*Neisseria gonorrhoeae* CHIM with 10 ^4^–10 ^5^ cfu inoculation at urethral site, treatment at end of observation period or development of symptoms	Hobbs & Duncan, Methods Mol Biology, 2019 ^15^ Duncan *et al.*, clinicaltrials.gov ^16^
*Streptococcus pyogenes* (Group A Strepto-coccus)	Healthy adults aged 18–40 years without risk factors for severe Group A Streptococcal disease	Pharyngeal, ~10 ^4^ cfu, 85% attack rate	Inpatient	5 days	Intramuscular benzathine penicillin 900mg single dose and oral rifampicin 300mg bd for 4 days at development of symptomatic GAS pharyngitis or day 5	Infectious upper respiratory pathogen with potential for systemic illness, treatment at end of observation period or development of symptoms. Novel pharyngitis clinical assessment grading score	Osowicki *et al.*, Lancet Microbe, 2021 ^31^
*Salmonella enterica* serovar. Typhi	Healthy adults aged 18–60 years, without prior typhoid vaccine or resident in typhoid endemic regions for >6 months	Ingestion, ~10 ^4^ cfu, 65% attack rate	Outpatient	14 days	Oral ciprofloxacin 500mg bd for 14 days at typhoid diagnosis or day 14	Infectious pathogen with potential for systemic illness, outpatient design with infection control procedures, treatment at end of observation period, development of symptoms (as well as bacteraemia)	Waddington *et al.*, Clin Infect Dis, 2014 ^36^
*Salmonella enterica* serovar. Paratyphi A	Healthy adults aged 18–60 years without prior typhoid vaccine or resident in enteric fever endemic regions for >6 months	Ingestion, ~10 ^3^ cfu, 60% attack rate	Outpatient	14 days	Oral ciprofloxacin 500mg bd for 14 days at paratyphoid diagnosis or day 14	Infectious pathogen with potential for systemic illness, outpatient design with infection control procedures, treatment at end of observation period, development of symptoms (as well as bacteraemia)	Dobinson *et al.*, Clin Infect Dis, 2017 ^37^; McCullagh *et al.*, BMJ Open, 2015 ^38^
Non-typhoidal *Salmonella (Salmonella enterica* serovar Typhimurium)	Healthy adults aged 18–50 years without prior *Salmonella* infection or typhoid vaccine	Ingestion, ~10 ^1^ – 10 ^6^, aim 60–75% attack rate	Inpatient (8 days) with subsequent outpatient phase (7 days)	14 days	Oral ciprofloxacin 500mg bd for 5–14 days at development of pre-specified symptoms or bacteraemia	Infectious pathogen with potential for systemic illness, dose escalation/de-escalation using continual reassessment model	Smith *et al.*, BMJ Open, 2024 ^39^

bd: bis die, twice daily; cfu: colony forming units; d: days; tds: ter die sumendum, three times daily.

## Study protocol

### Study synopsis

This study is a prospective dose-finding CHIM outpatient study which aims to establish the safety of an oropharyngeal gonorrhoea CHIM and identify the infectious dose required for healthy adult male participants to develop oropharyngeal *N. gonorrhoeae* infection following direct oropharyngeal application of *N. gonorrhoeae* strain AUSMDU00053933. A predicted total of approximately 20 to 35 eligible and consenting healthy male volunteers who exclusively have sex with people assigned male (assigned at birth) will be included in the study. Participants will be treated with ceftriaxone antimicrobial therapy for a maximum of 5 days after inoculation, undergo testing to confirm cure 7 and 14 days after treatment and will be followed up for 90 days after inoculation. Participant screening and recruitment is anticipated to commence in February 2026 and the study is anticipated to conclude in September 2026. This manuscript outlines the provisional protocol, version 1 (published October, 2025).

### Community engagement

Community engagement activities, including a qualitative research study, have been conducted throughout the protocol design stage, resulting in the incorporation of insights from key stakeholders. This qualitative research comprised of one focus group and 27 semi-structured interviews conducted between July and November, 2024, with a broad range of stakeholders. Participants included two community-based organization representatives, eight subject matter experts including clinical infectious diseases, sexual health, public health physicians and industry professionals, and a sample of 22 participants who would be eligible for participation in the study (i.e., 18 to 50 year old MSM without chronic medical illness) recruited through university, sexual health and community organization study advertisements. These qualitative data will be published elsewhere ^26^. A Clinical Trial Reference Committee including representatives from community-based organizations, a bioethicist, and infectious diseases experts will provide ongoing governance to maximize the ongoing acceptability of the trial. A process evaluation will be conducted in parallel with the CHIM to assess the experience of participants and to inform iterative improvement of the design and conduct of future trials ( CURE-NG Participant Questionnaire).

### Study objectives and outcomes

Study objectives and outcomes are summarised in Table 2. The primary objectives of this study are to establish i), the safety and tolerability of *N. gonorrhoeae* AUSMDU00053933 inoculation at the oropharynx; and ii), the dose of *N. gonorrhoeae* AUSMDU00053933 required to cause a reproducible microbiologically-confirmed *N. gonorrhoeae* oropharyngeal infection rate of 60 to 80% within five days of direct application by swab to the oropharynx. The secondary objectives of this study are to identify i) the proportion of participants at each dose level who develop symptomatic gonorrhoea pharyngitis; ii) the occurrence of severe, complicated or disseminated gonococcal infection among participants; and, iii) infection with *N. gonorrhoeae* at other mucosal sites during the study period. Exploratory objectives of this study include assessment of the microbiological characteristics and host immune responses of experimental human *N. gonorrhoeae* oropharyngeal infection, pharmacometric assessment of ceftriaxone treatment and acceptability of the study among participants.

**Table 2.** Primary and secondary objectives of the first oropharyngeal gonorrhoea controlled human infection model.

	Objectives	Outcomes
**Primary objectives**	1. To define the safety and tolerability of oropharyngeal inoculation of healthy volunteers with AUSMDU00053933 *N. gonorrhoeae*	Occurrence of solicited and unsolicited adverse events (as per the National Cancer Institute (NCI) Common Terminology Criteria for Adverse Events (CTCAE) within the study period
	2. To establish the dose (defined in colony forming units per millilitre of the dose vial) of AUSMDU00053933 *N. gonorrhoeae* required to cause reproducible oropharyngeal infection rate of 60–80% within 5 days	Oropharyngeal *N. gonorrhoeae* infection is defined as: 1) Microbiologically-confirmed oropharyngeal *N. gonorrhoeae* (defined as detection of *N. gonorrhoeae* by NAAT using two different *N. gonorrhoeae* genomic targets from a combined posterior oropharynx, palatine tonsils and saliva swab) >48 hours after challenge strain inoculation, or 2) Microbiologically-confirmed symptomatic *N. gonorrhoeae* pharyngitis, defined as sore throat and examination score ≥ 2 or grade 3 pharyngitis ( Figure 2) within 48 hours of inoculation of the challenge strain;
**Secondary objectives**	1. To identify the proportion of participants at each dose level who develop symptomatic gonorrhoea pharyngitis	Number of participants at each dose level who develop microbiologically-confirmed symptomatic *N. gonorrhoeae* pharyngitis
	2. To identify the occurrence of severe, complicated or disseminated gonococcal infection among participants	Identification of any participants that develop severe, complicated or disseminated gonococcal infection, defined as: 1) The detection of *N. gonorrhoeae* at sites other than the oropharynx and a clinical syndrome compatible with severe, complicated or disseminated gonococcal disease, or 2) A positive blood culture for *N. gonorrhoeae*.
	3. To assess for infection with *N. gonorrhoeae* at other mucosal sites during the study period	Detection of *N. gonorrhoeae* from other susceptible mucosalsites (rectal swab and first pass urine) from study participants during the study period via NAAT and/or culture
**Exploratory objectives**	To assess microbiological characteristics of experimental *N. gonorrhoeae* oropharyngeal infection	Microbiological detection (including qualitative culture and NAAT), phenotypic assessment (including presence of piliation), quantitation (including semi-quantitative culture, semi-quantitative (using the qualitative NAAT cycle threshold value) and quantitative NAAT (PCR)) and viability (by measuring the RNA-to-DNA ratio of a diagnostic target detected using NAAT) from throat swab during study period Genomic characterization of *N. gonorrhoeae* identified during study period Oropharyngeal microbiome characterization during the study period
	To assess the immune responses in healthy volunteers following experimental *N. gonorrhoeae* oropharyngeal infection	Changes in serological antibody responses during the study period Changes in mucosal antibody responses detected in saliva specimens during the study period Changes in systemic and oropharyngeal cytokine profile during the study period Changes in systemic cellular and mucosal response during the study period
	To assess pharmacometrics of intramuscular ceftriaxone for treatment of oropharyngeal gonorrhoea	Concentration of ceftriaxone in plasma and saliva after treatment and correlation of pharmacometric response with microbiological clearance
	To assess the motivations for participation and acceptability of participating in the oropharyngeal gonorrhoea CHIM	Identification of the motivations for participation; and acceptability of participating in the oropharyngeal gonorrhoea CHIM based on responses to a questionnaire completed before and after *N. gonorrhoeae* inoculation

NAAT: nucleic acid amplification test; PCR, polymerase chain reaction.

### Study recruitment

Several recruitment modalities will be employed to identify appropriate study participants. This will include advertising through local sexual health services and broader advertisement through higher education institutions, community-based organizations, social media and relevant websites. Advertising material used in this study will be co-designed with community-based organization representatives to maximize acceptability among potential participants. Participants will be offered reimbursement for their time for participation in the study.

### Eligibility criteria

Consenting healthy adults assigned male at birth, aged 18 to 50 years without pre-existing risk factors for severe disease will be recruited as study participants. Strict eligibility criteria have been designed to mitigate the risk of severe disease (eg, exclusion of individuals with complement deficiency, eculizumab use, immunocompromise including HIV, diabetes, drug/alcohol misuse ^40, 41^) and transmission to individuals at risk of severe disease (eg, exclusion of household contacts of immunocompromised) ^17^ ( Table 3). Detailed screening will include medical history, physical examination, baseline blood borne virus (BBV)/STI screening and blood tests. Individuals with a history of gonorrhoea infection in the preceding three months or who have previously received a *Neisseria meningitidis* serogroup B vaccine (eg. 4CMenB) will be excluded due to potential cross-protective immune responses against *N. gonorrhoeae* ^42, 43^. Individuals with a history of gonococcal-active antimicrobial usage in the prior three months will be excluded due to possible increased risk of antimicrobial resistance in subsequent gonorrhoea infections ^44^, due to carriage of antimicrobial resistance determinants in the oropharynx. Those with oropharyngeal *N. meningitidis* carriage detected in the week prior to inoculation will also be excluded due to the potential for transfer of virulence factors from *N. gonorrhoeae* to *N. meningitidis* in the oropharynx ^45^.

**Table 3.** Eligibility criteria for participants in the oropharyngeal gonorrhoea controlled human infection model.

Inclusion criteria
Male (assigned at birth) aged ≥ 18 to ≤ 50 years old on the day of informed consent
Identifies as a person who has sex with people assigned male at birth and does not have sex with individuals assigned female at birth (past 12 months)
Proficient in English language
Able and willing to comply with all study requirements
Ability to read and understand the participant information, provide written informed consent to participate in the trial and demonstrate understanding of study requirements by passing a quiz
Provide written agreement to comply with infection control guidelines during the experimental gonococcal infection phase until the study team advise that *N. gonorrhoeae* eradication has been confirmed
Able and willing to abstain from the use of mouthwash from the day of screening until the end of the study
Exclusion criteria
History of any clinically important cardiac, endocrinologic, haematologic, hepatic, immunologic, metabolic, urologic, pulmonary, neurologic, dermatologic, psychiatric, renal or other major disease, as determined by the Investigator
History of hospitalization for illness within the six months prior to enrolment into study or major surgery within the 12 months prior to enrolment into study
History of severe infectious disease including i) requirement for hospitalization for intravenous antibiotics; ii) prior *N. meningitidis* infection such as meningococcal meningitis or meningococcal bacteraemia; iii) ocular or disseminated gonococcal infection
History of cancer (except adequately treated squamous cell or basal cell carcinoma of the skin >5 years prior)
Any known or suspected immunodeficiencies or impairment/alteration of immune function including: • Congenital or acquired immunodeficiency including complement deficiency, antibody deficiency, chronic granulomatous disease, HIV infection or asplenism • Receipt of any immunosuppressive therapy such as anti-cancer chemotherapy or radiotherapy within the preceding 12 months • Any known or suspected autoimmune disorders (mild autoimmune disorders, such as eczema, are not exclusionary and will be determined by the Investigator)
Presence of implants or prosthesis (e.g. artificial joints, pacemakers)
History or presence of current alcohol abuse (defined as regular alcohol consumption of more than 40g per day), illicit drug use, or any prior intravenous usage of an illicit substance
Significant acute or chronic infection within 14 days prior to inoculation that the Investigator deems may compromise participant safety
Clinically significant disease or any condition or disease that might affect drug absorption, distribution or excretion, e.g. gastrectomy
History of tonsillectomy or adenoidectomy
Prior history of *N. meningitidis* serogroup B (e.g. 4CMenB) vaccination
A history of confirmed *N. gonorrhoeae* infection at any other site (urogenital, anorectal or oropharyngeal) in the three months prior to inoculation, including at screening or pre-enrolment testing
Detection of *N. meningitidis* on oropharyngeal swab at screening or pre-enrolment testing
Ex-smoker with >10 pack/year smoking history or a current active smoker defined as having smoked a cigarette or cigar in the four weeks prior to challenge
Any use of gonococcal-active antimicrobial therapy in the three months prior to inoculation
Any vaccination within the 28 days prior to challenge
Participation in a research study that involves blood sampling of more than 450ml/unit of blood, received or donated blood, blood products and/or plasma derivatives or any parenteral immunoglobulin preparation within three months before study, plans for donation at any time during the study and up to three months after the last blood test
Any clinically significant abnormal finding on laboratory screening investigations, with specific exclusion criteria including: • Serum creatinine level >1.1x upper limit of normal (ULN) and deemed clinically significant by the study physician • Serum ALT level >1.25x ULN and deemed clinically significant by the study physician • White blood count (WBC) <2.5 or >15.0 x 109/L and deemed clinically significant by the study physician • Haemoglobin level <110 g/L or above ULN and deemed clinically significant by the study physician • 50% complement haemolytic activity (CH50) outside normal limits • Positive serologic results for human immunodeficiency (HIV) antibodies, hepatitis B surface antigen (HBsAg), and/or hepatitis C virus (HCV) antibodies (+ PCR positive if Hepatitic C virus antibodies detected), syphilis serology (with evidence of active untreated infection) • A positive urine drug test at screening or pre-enrolment (e.g. amphetamines, barbituates, benzodiazepines, cannabinoids, cocaine, and opiates) unless there is an explanation acceptable to the Investigator (e.g. the participant has stated in advance that they consumed a prescription or over the counter product which contained the detected drug) and the participant has a negative urine drug screen on repeat testing • A positive alcohol breath test In the event of abnormal test results, confirmatory repeat tests will be requested
Known hypersensitivity or other contraindications to the use of cephalosporins, carbapenems or macrolides (including treatment with other medications that are contraindicated with ceftriaxone, azithromycin and ertapenem and these antimicrobials and cannot be safely withheld)
Participation in another research study involving an investigational product or other intervention within 12 weeks prior to enrolment, at any time during the study and up to 12 weeks after completion of the study
Use of any systemic immunomodulatory treatment including eculizumab, corticosteroids (topical corticosteroids acceptable), anti-inflammatories (beside sporadic use of non-steroidal anti-inflammatory drugs), anticoagulants (aspirin acceptable), investigational products, interleukins, interferons or growth factors within the previous three months, or anticipated use of such drugs during the study period.
Known hypersensitivity to soya protein or any other component of the liquid culture media used for inoculation
Use of inhaled or intranasal corticosteroid from 14 days prior to inoculation until 28 days after inoculation, or confirmation of *N. gonorrhoeae* eradication, whichever is later
Use of non-prescription drugs and herbal supplements (such as St John’s Wort) within 14 days or 5 half-lives (whichever is the longer) prior to inoculation. Use of vitamin supplements taken at standard doses is allowed.
Any other significant disease or disorder, which, in the opinion of the investigator, may either put the participants at risk because of participation in the study, or may influence the results of the study, or the participant’s ability to participate in the study
Intolerance of throat swab procedure (exaggerated gag reflex)
Occupational, household or intimate contact with immunocompromised individuals (including HIV infection, asplenia, malignancy, recurrent, severe infections and chronic immunosuppressant medication within the past 6 months)
Occupational or household contact with individuals <18 years of age
Residence in unstable or emergency housing
Any employee of the sponsor or research site personnel directly affiliated with this study or their immediate family members defined as spouse, parent, sibling or child whether biologic or legally adopted

Only individuals assigned male at birth who exclusively have sex with people assigned male at birth will be recruited, due to the risk of acute and long-term reproductive health complications of urogenital gonorrhoea on the female reproductive system ^6^. This population of adult MSM are a priority population to include in research exploring *N. gonorrhoeae* treatment and prevention strategies, as there is a high prevalence of *N. gonorrhoeae* infection in MSM both in Australia ^46^ and other high-income settings worldwide ^1^. Although the oropharynx is the only site being inoculated with *N. gonorrhoeae* in this study, there is a risk of autoinoculation ^47, 48^ or transmission to others via contact with the mouth or saliva ^49^. As such, individuals with a female reproductive system and those with household or occupational contact with immunocompromised individuals or individuals < 18 years of age will be excluded ^17^. Only prospective participants who are willing and able to adhere to study procedures to mitigate the risk of transmission of *N. gonorrhoeae* to others will be included. Specific infection control procedures will be included in participant information and informed consent materials ( Table 4). Participants will also be required to demonstrate understanding of the study procedures and requirements before participating in the study. Due to increased risk of disseminated gonococcal infection described in some studies ^40, 50, 51^, people living with HIV (PLHIV) will be excluded from this initial study.

**Table 4.** Infection control guidelines for study participants in the oropharyngeal gonorrhoea controlled human infection model.

Day of inoculation at clinical trials centre
• Participants must wear a surgical mask at all times unless within their personal room until discharge from the clinical trials centre
• Participants must not enter the personal rooms of other participants
• Participants must remain in the clinical trials centre for the duration of the inoculation procedure and a 1 hour monitoring period thereafter
• Participants must wash their hands with soap for 30 seconds or use alcohol based hand rub before leaving their room
• Participants must not have contact with known immunosuppressed individuals
• Participants must not have any contact that could involve saliva or respiratory secretions to others during the clinical trials centre attendance
From inoculation to confirmed *N. gonorrhoeae* eradication
From inoculation to confirmed *N. gonorrhoeae* clearance:
• Participants must not engage in sexual activity including contact with the mouth (including deep kissing and saliva use in sexual activity), or contact with the penis, urine, semen or anorectal region with any other individual
• Participants must not have any contact with any other individual that has a high risk of transmission of saliva, including:
○ Sharing objects placed in the mouth such as cutlery or drinking vessels
○ Sharing of sex toys
• Participants must not use illicit drugs or alcohol
• Participants must avoid contact with known immunosuppressed individuals and individuals 18 years of age
• Participants must wash their hands with soap for 30 seconds or use alcohol based hand rub after contact with their own mouth or saliva

Study-specific exclusion criteria focus on mitigating the risk of participants developing complications and on reducing the risk of bias related to primary, secondary or exploratory outcomes. These include a history of disseminated gonococcal infecion; complement deficiency or other known or suspected congenital or acquired immunodeficiency; history of tonsillectomy; intolerance of throat swab procedure (e.g. exaggerated gag reflex) and known hypersensitivity or contraindication to b-lactam antimicrobials, soya protein (included in the bacterial culture medium) or other constituent in the bacterial culture medium ( Table 5). Exclusion criteria include restriction of specific concomitant medications in the month prior to inoculation until confirmed cure, specific vaccines, systemic and intranasal corticosteroids, immunomodulators and anti-inflammatory therapy.

**Table 5.** Constituents in the bacterial culture media.

Agar	L-glutamine	Sodium hydroxide
Ammonium bicarbonate	L-ornithine	Sodium lactate
Corn starch	Nicontinamide adenine dinucleotide	Soya peptone
Di-potassium hydrogen phosphate	Oxaloacetate	Spermidine
Ferric nitrate	Peptone	Thiamine hydrochloride
Glucose	Potassium dihydrogen phosphate	Thiamine pyrophosphate
Glycerol	Sodium acetate	Uracil
Hydrogen chloride	Sodium bicarbonate	
Hypoxanthine	Sodium chloride	

### Infection control considerations

Participants will be managed using infection control protocols observing contact and droplet precautions in the trial centre on the day of inoculation. As *N. gonorrhoeae* is culturable from the saliva of individuals with oropharyngeal gonorrhoea ^52, 53^ and transmission via fomites has been reported ^54^, contact and droplet precautions have been adopted to mitigate risk to trial personnel. Because the inoculation procedure may induce coughing due to a gag reflex, a more stringent infection control protocol including droplet precautions has been instituted for the day of inoculation for this study. Participants will be managed in single-rooms on the day of inoculation at the trial centre. All high-touch surfaces will be decontaminated after patient discharge from the clinical trials unit as per local policy.

Study participants will receive clear verbal and written instruction regarding infection control procedures in informed consent materials ( Table 4), and be instructed to adhere to these instructions until *N. gonorrhoeae* eradication is confirmed. Information sheets, detailing access to free gonorrhoea testing and treatment services, will be provided to the participants that can be given to any contacts in the case that infection control procedures have been breached during the relevant period (from inoculation until confirmed eradication). In the event that participants advise study staff of infection control breaches during the relevant period (inoculation until confirmed eradication), study staff will also facilitate contact tracing, including anonymous notification of *N. gonorrhoeae* exposure and referral to free testing and treatment services. As gonorrhoea is a notifiable disease in Australia, the jurisdictional public health department have also been notified of the study.

### Study procedures and schedule

**Figure 1.**
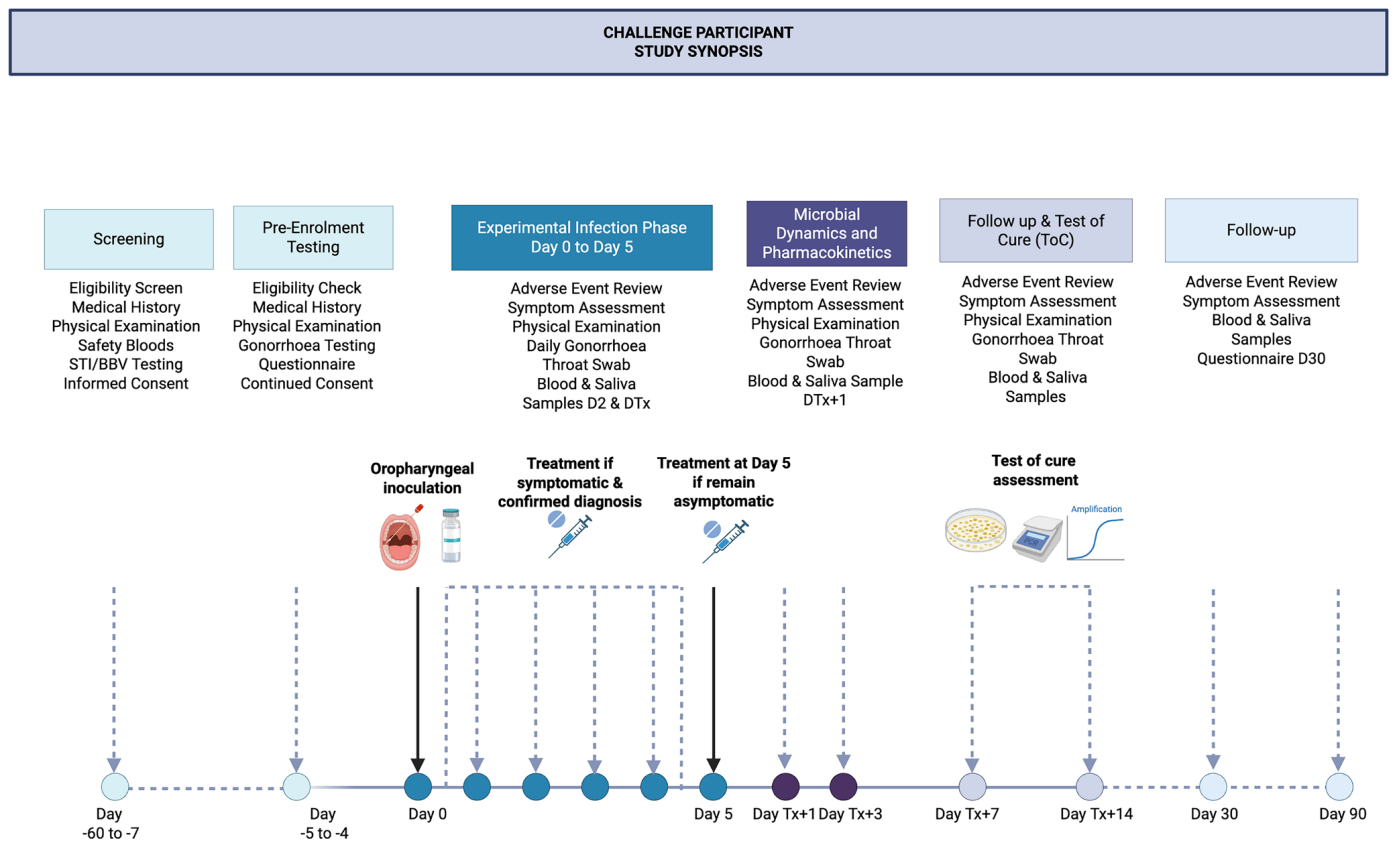
1. Overview of participant timeline from screening to study completion. DTx, day of treatment; BBV, blood borne virus; STI, sexually transmitted infection; ToC, test of cure. Created in BioRender. Williams, E. (2025) https://BioRender.com/w9c1nrb.

Study procedures and schedule of events are described in Figure 1 and Table 6, respectively. Study participants will be screened for eligibility between two months and five days (day -60 to day -5) prior to enrolment. Study participants will undergo pre-enrolment screening on day -5 to day -4 to confirm eligibility prior to enrolment in the study on day 0.

**Table 6.** Study procedures for participants for the oropharyngeal gonorrhoea controlled human infection model.

Schedule of Activities															
Procedures	Phone Screening	Screening	Pre-enrolment Testing	Experimental Infection Period			Follow-up Visits						Persistent Carriage Confirmation	Treatment for Persistent Carriage	Eradication Testing After Treatment for Persistent Carriage
Study Day	D-60 to D-7	D-60 to D-7	D-5 (+/-1)	Challenge: D0	Daily Monitoring (D1 to D4) ^a^	Symptomatic Infection / Treatment (DTx) ^b^	DTx +1	DTx+3	DTx +7: Test of cure (+/-1)	DTx+14 ^c^: Test of cure (+/-1)	D30 (+/-3)	D90 (+/-5)	+7D after initial positive test of cure (+/-1)	Within 24hrs of lab notification of confirmed persistent carriage	+7, +14, and +21 ^d^ days after treatment for persistent carriage (+/-1)
Day of Week			Tue	Sun	Mon-Thu	Mon-Fri					Tue	Sat			
**Consent, history and examination**															
Review eligibility	X	X	X	X											
Verbal informed consent	X														
Written informed consent		X													
Confirmation of consent			X	X											
Demographics	X	X													
Medical history	X	X	X	X											
Social history	X	X	X	X											
Concomitant medications		X	X	X	X	X	X	X	X	X	X	X	X	X	X
Symptom check					X	X	X	X	X	X	X	X	X	X	X
Weight & height		X													
Vital signs		X	X	X	X	X	X	X	X	X	X		X	X	X
Comprehensive clinical examination		X													
Targeted examination			X	X	X	X	X	X	X	X	X		X	X	X
Symptom-directed physical examination					X	X	X	X	X	X	X				
Adverse events				X	X	X	X	X	X	X	X	X	X	X	X
**Clinical tests**															
Alcohol breath test		X	X	X											
Urine drug screen		X	X												
Hematology blood tests		X				X							X		
Biochemistry blood tests		X				X							X		
CH50 determination		X													
Serum (blood test) for storage				X											
Serology (blood test) for immunological assessment				X	X ^i^	X			X	X	X	X			
PBMCs (blood test) for immunological assessment				X	X ^i^				X		X	X			
Plasma for drug levels						X	X								
BBV Serology (HIV Ag/Ab, HBsAg, HCV Ab, syphilis Ab)		X													
ECG													X		
Combined oropharyngeal swab (CT/NG PCR; culture)		X	X	X	X	X	X	X	X	X			X	X	X
Combined oropharyngeal swab (viability assessment)						X	X	X	X	X			X		X
Nasopharyngeal swab for immunology				X					X		X	X			
Saliva (immunological assessment)				X	X ^i^	X			X	X	X	X			
Saliva (microbiological assessment)				X	X ^i^	X			X	X	X	X			
Saliva (drug levels)						X	X								
Rectal swab CT/NG PCR ^e^		X	X	X		X ^f^			X ^f^	X ^f^			X		X
Urine CT/NG PCR ^e^		X ^g^	X ^g^	X ^g^		X ^f^			X ^f^	X ^f^			X		X
Blood culture						X ^f^									
**CHIM procedures**															
Photo of oropharynx				X		X			X	X			X		X
Review infection control adherence					X	X	X	X	X	X			X	X	X
Participant experience quesetionnaire			X								X				
Inoculation				X											
Antibiotic treatment ^h^						X								X	

^a^ Daily monitoring between Inoculation and Symptomatic Infection/Treatment visits. If Symptomatic Infection occurs earlier than D5, this will replace and stop the Daily Monitoring Visits. ^b^ DTx = Day of Treatment. This visit will not occur any later than D5. ^c^ Day Tx+14 will not be undertaken if the participant has remained uninfected with oropharyngeal gonorrhoea ^d^ +21 timepoint will not be required if test of cure returns negative at +14 timepoint ^e^ Self-collected sample ^f^ If *N. gonorrhoeae* infection is established ^g^ First pass urine collection ^h^ Treatment may also be provided at any time upon participant request ^i^ Day 2 only Ab, antibody; Ag/Ab, antigen/antibody; BBV, blood borne virus; CH50, total complement activity; CHIM, controlled human infection model; CT, *chlamydia trachomatis*; ECG, electrocardiogram, HIV, human immunodeficiency virus; HBsAg, Hepatitis B surface antigen; HCV, hepatitis C virus; NG, *Neisseria gonorrhoeae*; PCR, polymerase chain reaction

After confirmation of informed consent by the investigator or delegated study team member, eligibility and collection of baseline blood for exploratory immunological assessment, inoculation will take place on day 0 at the clinical trials centre. Building on established oropharyngeal inoculation procedures from the *Streptococcus pyogenes* CHIM, each participant will undergo a single inoculation, delivered by applying *N. gonorrhoeae* from a thawed single-dose vial to the oropharynx using a sterile swab ^55^. Inoculation will take place in a dedicated room at the clinical trials facility. The designated single-dose vial will be thawed from storage at ≤-70°C, and inoculated within 0 to 10 minutes of thawing. Participants will be required to fast (inclusive of all food and drink) for 90 minutes before and after inoculation. The participant will be prepared by being seated in a semi-recumbent position. The vial will be prepared by performing gentle inversion ten times to mix the inoculum and the sterile swab will be dipped in the vial for ten seconds. The participant will be instructed to tilt their head backwards and open their mouth widely. Using a tongue depressor to hold the tongue in place, the operator will apply the inoculum by rolling the swab back and forth over the tonsillar arches and posterior oropharynx. After inoculation, the single-dose vial will be recapped, marked as used with the time, date of inoculation and study participant identification number, and returned to the laboratory for storage. Study monitoring procedures will track all doses received, dispensed, inoculated and returned to pharmacy and these data will be reconciled at the end of the study.

**Figure 2.**
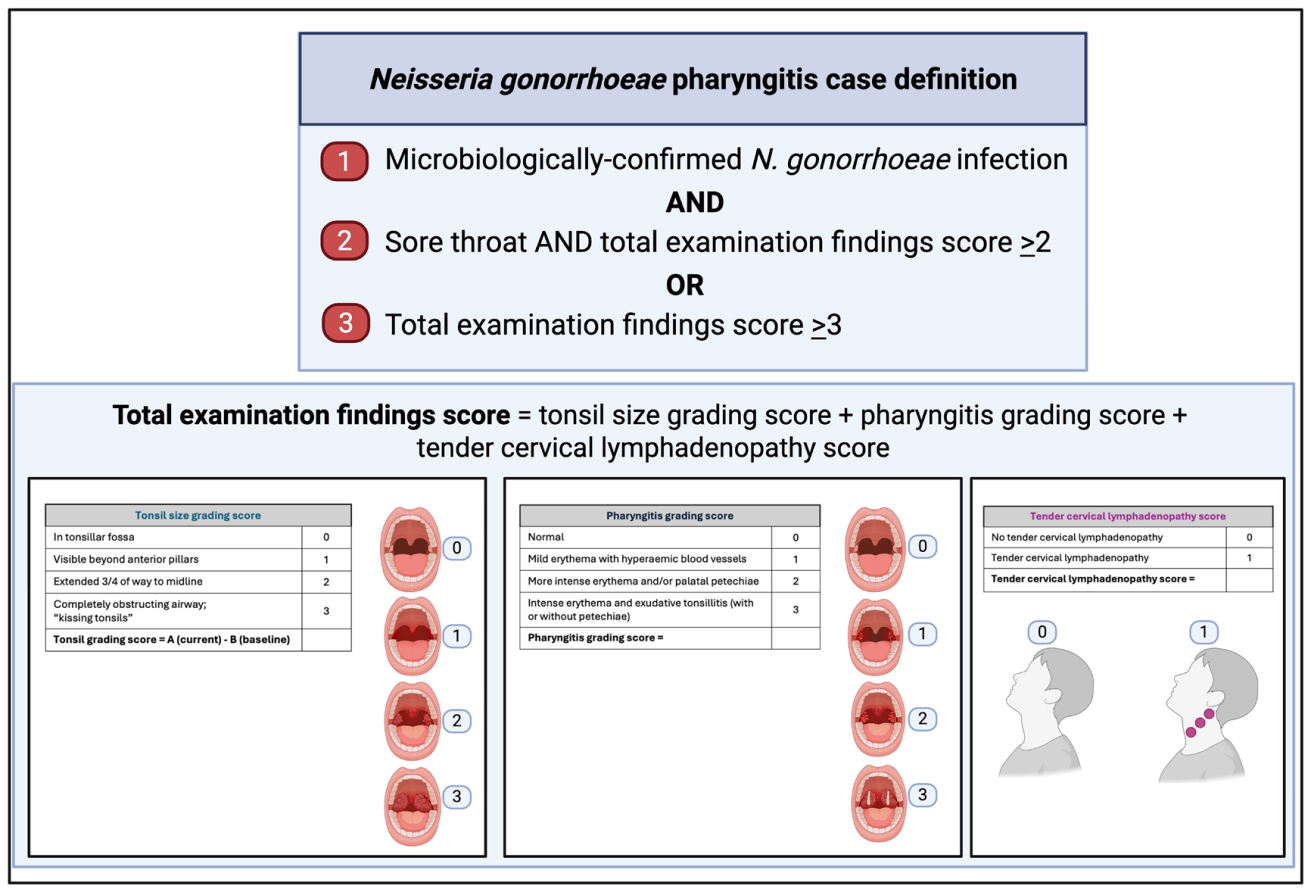
*Neisseria gonorrhoeae* pharyngitis case definition. Created in BioRender. Williams, E. (2025) https://BioRender.com/c61x06a.

Participants will be observed in the clinical trials unit for one hour after inoculation, and attend the trial unit daily for up to five days after inoculation. As oropharyngeal gonorrhoea is predominantly an asymptomatic infection with limited clinical impact, an oropharyngeal gonorrhoea CHIM can be safely performed in the outpatient setting with risk mitigation procedures in place. As transmission is expected to be negligible with appropriate infection control procedures in place and there is a high prevalence of oropharyngeal gonorrhoea in the target recruitment population of MSM in Melbourne, inpatient admission and confinement is not considered necessary for this CHIM. The prevalence of oropharyngeal gonorrhoea in a cross-sectional study of 3,677 MSM in Melbourne in 2016-2017 was 6.2% ^56^; and oropharyngeal gonorrhoea prevalence was up to 16% in a prospective cohort study of 100 MSM with increased risk of oropharyngeal gonorrhoea (including HIV PrEP usage or oropharyngeal gonorrhoea infection in the prior three months) in Melbourne in 2019 ^57^. Daily visits will include review of adverse events, participant symptoms and sexual activity/personal contact activities, physical examination, recording of vital signs and collection of a combined oropharyngeal swab (collected from the posterior oropharynx, palatine tonsils and saliva) ( Figure 1). Participants will be treated with 1g ceftriaxone by intramuscular injection in the following circumstances: i) at day five after inoculation; ii) within 24 hours if they develop symptomatic pharyngitis, defined as sore throat and examination score ≥2 or grade ≥3 pharyngitis ( Figure 2) with microbiologically-confirmed *N. gonorrhoeae* infection; or, iii) upon participant request.

On the day of treatment, blood for haematology, biochemistry and exploratory immunological assessment will be collected. Blood cultures, first pass urine and rectal swabs for *N. gonorrhoeae* NAAT will be collected from individuals in whom oropharyngeal gonorrhoea infection has been established. Participants will attend an outpatient review on days 1, 3, 7 and 14 after antimicrobial therapy for clinical assessment and microbiological sampling including test of cure (combined oropharyngeal swab for gonococcal culture and *N. gonorrhoeae* NAAT). Study participants will also attend an outpatient review at one and three months after inoculation for clinical assessment to assess for the development of post-infection complications and undergo further sampling for immunological assessment. Supportive therapy will be provided as required for symptom control, however most infections are expected to be asymptomatic. Throughout the trial, participants will have access to 24-hour telephone support and advice from clinical trials staff, including medical personnel, and access to urgent review and sepsis treatment through the partner adult tertiary hospital in the unlikely event that acute infectious complications occur.

### Outcome measures

As this is a first-in-human experimental oropharyngeal gonorrhoea infection study, it has two primary outcomes, one being safety and tolerability and the other dose-finding of the *N. gonorrhoeae* challenge strain, AUSMDU00053933, at this anatomical site. The occurrence of serious adverse events and solicited and unsolicited adverse events within the study period will be monitored by the Trial Management Group (TMG) overseen by the medical monitor and Safety Monitoring Committee (SMC) throughout the study to establish the safety of the CHIM.

Oropharyngeal gonorrhoea is predominantly an asymptomatic infection. As such, the case definition of oropharyngeal *N. gonorrhoeae* infection utilized to establish the dose of AUSMDU00053933 required to cause a reproducible oropharyngeal infection rate of 60-80% within five days for this study can be established via microbiological criteria alone, or combined clinical and microbiological criteria. This case definition includes i) microbiologically-confirmed oropharyngeal *N. gonorrhoeae* >48 hours after challenge strain inoculation; or, ii) microbiologically-confirmed symptomatic *N. gonorrhoeae* pharyngitis, defined as sore throat and examination score ≥2; or grade ≥3 pharyngitis ( Figure 2) within 48 hours of inoculation of the challenge strain.

Microbiologically-confirmed *N. gonorrhoeae* will be defined as detection of *N. gonorrhoeae* by NAAT with two different *N. gonorrhoeae* genomic targets from a combined oropharyngeal swab collected from the posterior oropharynx, palatine tonsils and saliva. *N. gonorrhoeae* NAAT will be performed using the commercial multiplex Xpert CT/NG (Cepheid, Sunnyvale, CA) polymerase chain reaction (PCR) which has two *N. gonorrhoeae* targets that identify the presence of non-contiguous highly conserved chromosomal DNA regions in *N. gonorrhoeae*. The case definition for clinical *N. gonorrhoeae* pharyngitis used in this study was developed for use in the *S. pyogenes* CHIM ^55^ and is adapted from the Centor and McIsaac scores for prediction of streptococcal pharyngitis ^58^ ( Figure 2). Although this score has not been validated for use for oropharyngeal gonorrhoea, it has been successfully implemented in the *S. pyogenes* CHIM ^31^ and provides a measure of pharyngeal inflammation including tonsillar size change, pharyngitis grading and cervical lymphadenopathy, which can be used to document and grade physical examination findings in conjunction with symptom assessment and microbiological testing. 

Participants will have daily combined oropharyngeal swab collected from the posterior oropharynx, palatine tonsils and saliva for *N. gonorrhoeae* NAAT and culture during the experimental gonorrhoea infection phase. If the participant develops symptoms and signs consistent with pharyngitis, additional samples will be collected to facilitate testing for other common causes of pharyngitis in the community, including a nasopharyngeal swab for respiratory viruses and throat swab for *S. pyogenes.* These tests will be performed if *N. gonorrhoeae* is not detected on NAAT. In the event of asymptomatic microbiologically-confirmed *N. gonorrhoeae* infection, participants will be monitored daily in the clinical trials centre and treated on day five after inoculation. In the event that no infection has occurred by day five, a final combined oropharyngeal swab will be collected, followed by appropriate curative antimicrobial therapy for *N. gonorrhoeae* infection. Although *N. gonorrhoeae* culture will be performed concurrently with all NAAT sampling on throat swabs, results of these tests will not inform the primary microbiological outcome of the study, as *N. gonorrhoeae* culture for detection of *N. gonorrhoeae* infection is significantly less sensitive than NAAT at the oropharynx ^59^. In observational studies, NAAT has been shown to be approximately five-fold more sensitive than culture ^52^. Secondary and exploratory outcomes include clinical, microbiological, immunological, pharmacometric and process evaluation outcomes ( Table 2).

### Antimicrobial therapy

All participants will receive antimicrobial therapy aimed at eradicating the *N. gonorrhoeae* challenge strain from the oropharynx ( Table 7). Antimicrobial therapy will comprise of single-dose intramuscular ceftriaxone 1000mg in 3.5mL of 1% lignocaine. This primary treatment regimen aligns with United States Center for Disease Control and British Association for Sexual Health and HIV recommendations for treatment of oropharyngeal gonorrhoea ^60, 61^. Although it does not align with Australian STI treatment guidelines, where dual therapy with 500mg intramuscular ceftriaxone and 2g oral azithromycin is recommended ^62^, it has been selected to align with local health service guidelines and reduce the probability of gastrointestinal adverse events associated with azithromycin therapy ^63^. It also avoids the administration of two classes of broad-spectrum antimicrobial therapy to trial participants. The *N. gonorrhoeae* challenge strain is susceptible to all clinically-relevant anti-gonococcal antimicrobials (i.e ceftriaxone, azithromycin, ciprofloxacin and tetracycline).

**Table 7.** Indications for antimicrobial therapy for participants during the oropharyngeal gonorrhoea CHIM.

Indications for Antimicrobial Therapy	Criteria	Description
Study Participants		
Clinical symptoms/signs due to *N. gonorrhoeae* pharyngitis	As soon as practicable and within 24 hours of onset *of N. gonorrhoeae* pharyngitis	*N. gonorrhoeae* pharyngitis defined as: i) sore throat, examination score ≥2 ( Figure 2) and microbiologically confirmed *N. gonorrhoeae* infection OR ii) examination score ≥3 ( Figure 2) and microbiologically confirmed *N. gonorrhoeae* infection
End of experimental infection phase	At day 5 for all study participants, regardless of *N. gonorrhoeae* infection status, unless treated earlier due to symptomatic *N. gonorrhoeae* infection or participant request	Treatment provided at end of 5-day experimental infection phase, regardless of *N. gonorrhoeae* infection status
Participant request	As soon as practicable and within 24 hours of request by the participant, regardless of symptoms or signs	Treatment provided upon participant request at any time

### Test of cure and additional antimicrobial therapy

Test of cure to confirm *N. gonorrhoeae* eradication will be performed for all participants who develop *N. gonorrhoeae* infection during the study. Test of cure will be undertaken by performing a *N. gonorrhoeae* culture and NAAT on a combined oropharyngeal swab collected from the posterior oropharynx, palatine tonsis and saliva on day 7 post antimicrobial therapy and confirmed by performing a *N. gonorrhoeae* culture and NAAT on combined oropharyngeal swab on day 14 post antimicrobial therapy. It is expected that *N. gonorrhoeae* culture will be negative at day 7 post antimicrobial therapy and *N. gonorrhoeae* NAAT will be negative at day 14 post antimicrobial therapy. If *N. gonorrhoeae* is not detected at these timepoints, *N. gonorrhoeae* challenge strain eradication has been confirmed. If *N. gonorrhoeae* is detected at these timepoints, the following additional testing will be performed to confirm persistent oropharyngeal carriage prior to the administration of additional antimicrobial therapy.

If *N. gonorrhoeae* is identified by culture of a combined oropharyngeal swab on day 7 after antimicrobial therapy, phenotypic antimicrobial susceptibility testing and whole genome sequencing will be performed to assess the cause of potential treatment failure (i.e., *in vivo* development of resistance to the challenge strain or infection with an alternative *N. gonorrhoeae* strain), and repeat *N. gonorrhoeae* culture and NAAT will be performed on a combined swab collected on day 14 days after antimicrobial therapy to confirm persistent infection. If *N. gonorrhoeae* is identified by culture at this time, individuals will be managed as a suspected case of persistent oropharyngeal *N. gonorrhoeae* infection and further antimicrobial therapy will be provided as below.

It is expected that *N. gonorrhoeae* will not be detected on a combined oropharyngeal swab via NAAT for the majority of participants at day 14. However, as *N. gonorrhoeae* DNA can be detected by NAAT in up to 8% of cases 14 days after treatment ^64^, if *N. gonorrhoeae* is detected by NAAT at this timepoint, repeat sampling will be performed on day 21 after treatment, when detection of persistent *N. gonorrhoeae* by NAAT after successful treatment is less likely ^65^. Phenotypic antimicrobial susceptibility testing and whole genome sequencing will be performed on any available culture isolates from the participant to assess the cause of potential treatment failure (e.g. development of AMR), assess if there has been a new infection (e.g. infection with a different strain) and to guide appropriate management. Repeat NAAT and culture will be performed at day 21 after treatment to assist determination of whether persistent *N. gonorrhoeae* detection by NAAT at the previous timepoint represented treatment failure, detection of non-viable DNA or new infection. If *N. gonorrhoeae* is not detected at this timepoint, *N. gonorrhoeae* challenge strain eradication has been confirmed. If *N. gonorrhoeae* remains detectable at this time, the case will be managed as a suspected case of persistent oropharyngeal *N. gonorrhoeae* infection and further antimicrobial treatment will be administered.

In the unlikely event of persistent *N. gonorrhoeae* infection, treatment will be guided by results of antimicrobial susceptibility testing, whole genome sequencing, and clinical judgement in consultation with the SMC. Options for treatment recommended by various international bodies will be considered: i) single-dose intramuscular ceftriaxone 1g in 3.5mL of 1% lignocaine plus one dose of oral azithromycin 2g, ii) daily intravenous ceftriaxone 1g administered for three days; and iii) daily intravenous ertapenem 1g administered for three days ^23^.

### Challenge strain

A novel *N. gonorrhoeae* challenge strain, AUSMDU00053933, collected from a 27 year-old male with symptomatic urethritis attending Melbourne Sexual Health Centre in 2017 was selected for this study. The strain’s multi-locus sequence type (MLST) is 1596 and its *N. gonorrhoeae* sequence typing for antimicrobial resistance (NG-STAR) is 4332. It is susceptible to all clinically-relevant antimicrobials (including ceftriaxone, azithromycin, ciprofloxacin, and tetracycline) ^27^. As previously described, a genomics-based selection strategy was used to shortlist this strain from 5,881 clinical isolates of *N. gonorrhoeae* collected from adult patients in Victoria, Australia between January 2017 and June 2021 ^27^. This strategy utilized clinical, phenotypic and genomic characteristics to shortlist strains of global clinical relevance that met stringent safety criteria to reduce the risk of disseminated gonococcal infection and clinically significant AMR ^27^. The results of *in vitro* assays and cell bank manufacture pilot studies, which will be published separately, indicated this strain is a suitable challenge agent for an initial oropharyngeal gonorrhoea CHIM, given its characteristics (attachment to epithelial cell lines, minimal epithelial cell invasion, absence of induction of a cytotoxic or inflammatory response, susceptibility to killing by normal human serum, and stability in cell bank manufacture).

### Dose selection

The dose required to cause oropharyngeal infection in 60 to 80% (ID _60_-ID _80_) of participants with AUSMDU00053933 is unknown. However, the estimated dose resulting in infection in 50% of participants (ID _50_) in the gonorrhoea urethritis model using alternative challenge strains has been calculated as 1.8 x 10 ^3^ colony forming units (CFU) for *N. gonorrhoeae* MS11mkC and 1.0 x 10 ^5^ CFU for *N. gonorrhoeae* FA1090 ^66^. As this study is aiming to define the ID _60_ to ID _80_, dosing will commence at approximately 10 ^4^ CFU, which approximates the estimated ID _60_ to ID _80_ of MS11mkC. This starting dose also approximates the predicted ID _70_ based on continual reassessment method (CRM) dose prediction simulations ( CURE-NG Continual Reassessment Model Design (Extended Data)). This dose is approximately 10-fold lower than the median gonococcal bacterial DNA load detected per throat swab and per millilitre of saliva of individuals with oropharyngeal gonorrhoea, respectively ^52^; approximately 10-fold lower than the median gonococcal bacterial DNA load detected per urethral swab of individuals with asymptomatic gonococcal urethritis, and approximately 100-fold lower than those with symptomatic gonococcal urethritis ^67^. *In vitro* studies suggest that the estimated volume of uptake and concentration of bacteria released from Dacron swabs is approximately 10-fold lower than the concentration of the single-use vial they are dipped in prior to inoculation ^68^. As such, to obtain a starting dose of approximately 10 ^4^ CFU, the swab will be inoculated in a single-use vial containing a concentration of 10 ^5^ ± 0.5 log _10_/mL Due to the uncertainties involved in calculating the actual delivered dose, this study will refer to the dose level as the concentration of the dose vial rather than the estimated dose delivered (i.e., starting dose, 10 ^5^ ± 0.5 log _10_ CFU/ml). This study has been designed based on a dose-escalation algorithm using a CRM as described below. Up to five dose levels are planned for testing if required (10 ^4^ ± 0.5 log _10_ CFU/mL to 10 ^8^ ± 0.5 log _10_ CFU/mL). 

### Continual Reassessment Model study design

**Figure 3.**
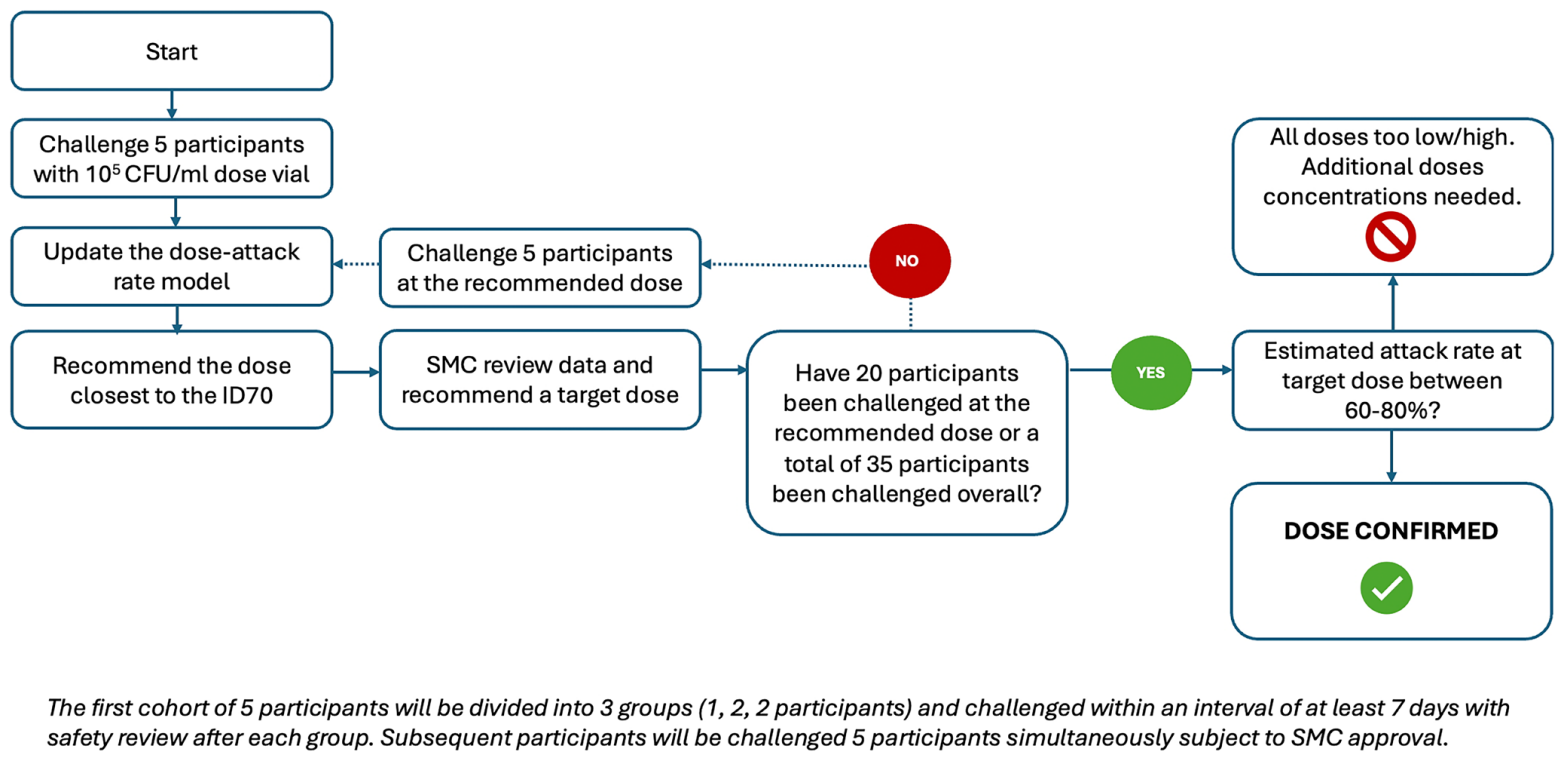
Overall study design and dose escalation/de-escalation procedures for the oropharyngeal gonorrhoea controlled human infection model. CFU, colony forming units; ID70, infectious dose 70%; ml, millilitre; SMC, Safety Monitoring Committee.

A CRM will be used to identify the AUSMDU00053933 dose that can establish oropharyngeal *N. gonorrhoeae* infection in 60–80% of participants ( Figure 3). Initially implemented in Phase I trials to identify the maximum tolerated dose of a new drug or treatment, this method has recently been employed in the design of dose-finding CHIMs ^39^. CRMs have been shown to more accurately identify the true maximum tolerated dose of a new therapy compared to traditional rule-based designs in Phase I trials ^69^.

The CRM study design was evaluated using a two-parameter dose-response model, within a Bayesian framework (described further in *Statistical Analysis*). Starting dose was specified as 10 ^4^ CFU (dose vial 10 ^5^ ± 0.5 log _10_ CFU/ml), and five individuals were allocated per group. Individual trials were simulated according to the following CRM rules:

1. Inoculate group at current dose.
2. Dose-response model is fit to observed data (i.e., number of infected individuals at current dose).
  ◦ If the model-based estimate of the ID _70_ is ≥2 log _10 _CFU above the current dose, escalate two log _10_ doses.

Otherwise,
  ◦ if <75% chance that posterior probability of infection at that dose >0.6, then escalate one log _10_ dose.
  ◦ if >95% chance that posterior probability of infection at that dose >0.8, then de-escalate one log _10_ dose.
  ◦ if >75% chance that posterior probability of infection at that dose >0.6, and <95% chance that posterior probability of infection at that dose > 0.8, maintain current dose.
3. Repeat steps 2–3 until 20 individuals have been inoculated at the target dose or 35 individuals have been inoculated in total.

Given the sparse information on the dose-response properties of AUSMDU00053933, prior distributions on model parameters were specified such that there was approximately uniform probability of the ID _70_ falling between 10 ^2^ and 10 ^7^ (( CURE-NG Continual Reassessment Model Design (Extended Data); .Figure S1). This range was informed based on existing CHIMs for similar pathogens ( Table 1). The starting dose vial of AUSMDU00053933 used in this study will be 10 ^5 ^± 0.5 log _10 _CFU/ml, and may increase to a maximum of 10 ^8^ ± 0.5 log _10 _CFU/ml and de-escalate to a dose of 10 ^4^ ± 0.5 log _10 _CFU/ml according to the CRM. The CRM dictates that the model is updated after each cohort of five participants completes the experimental infection phase, and subsequent dose allocation is informed by the model-based estimate. The dose closest to the target attack rate of 70% (ID _70_) will be identified using the CRM and presented to the SMC, who will recommend the dose for the subsequent cohort to the Trial Steering Committee (TSC) for approval. The next cohort of five participants will be challenged with the recommended dose until 20 participants have been inoculated at the dose predicted to achieve the target attack rate of oropharyngeal *N. gonorrhoeae* infection in 60–80% of participants or 35 participants have been inoculated in total ( Figure 3). Once the dose closest to the target attack rate of 70% (ID _70_) is identified using the CRM, results will be presented to the SMC, who will make a recommendation to the TSC for approval to close the study.

The operating characteristics of the CRM for this oropharyngeal *N. gonorrhoeae* CHIM are detailed in the extended data ( CURE-NG Continual Reassessment Model Design (Extended Data); Table S1-S2 and Figures S1-S5). In brief, 1000 prior simulations indicated that an average of 27.8 participants will be expected to be required to achieve stopping criteria, and 96.7% of simulated trials will have 20 participants inoculated at the target dose. The 90% credible intervals associated with the estimated ID _70 _for these simulated trials had an average width of 1.74 log _10 _CFU. Individual scenarios were evaluated where the underlying ID _70_ sits at the extremes, ranging from approximately 10 ^2.5^ – 10 ^6.8^ CFU (100 simulations at each) ( CURE-NG Continual Reassessment Model Design (Extended Data); Figures S1, S2). As expected, given the starting dose of 10 ^4 ^CFU (dose vial 10 ^5 ^± 0.5 log _10 _CFU/ml), the average sample sizes were higher where the ID _70_ was expected to be higher, ranging from 23.35 for 10 ^3.27 ^CFU to 30.15 for 10 ^6.8 ^CFU ( CURE-NG Continual Reassessment Model Design (Extended Data); Table S1; Figures S3-5). Similarly, the average width of the credible intervals of the ID _70_ estimate were wider where the underlying ID _70_ is expected to be higher, due to the shape of the dose-response curve ( CURE-NG Continual Reassessment Model Design (Extended Data); Table S2).

### Statistical analysis

After each group of 5 participants has completed the experimental infection phase at the dose designated by the CRM, the primary endpoint infection data will be analysed. Data analysis will be done in a Bayesian framework using Markov Chain Monte Carlo to characterise the posterior distributions of the model parameters. The dose-response relationship will be represented by a two-parameter, independent-action dose-response model. Specifically, the probability of infection, *P _inf_*, at dose *D*, is given by:


$$pinf(D)=1-{\left(1+\frac{D}{b}\right)}^{-a}$$

Where *b* and *a* are the dose-response model parameters. The number of individuals infected at a given dose then follows a binomial distribution, with number of trials given by the group size and probability of ‘success’ given by *P _inf_(D)*. After each group is inoculated and the analysis conducted, results will be reported to the SMC to determine appropriate course of action for subsequent groups, or stopping.

### Safety measures

Serious Adverse Events (SAE) will be monitored as per standard definitions throughout the study. Medically Significant Events specific to this study include: i) disseminated gonococcal infection, including (a) purulent arthritis, tenosynovitis-dermatitis-polyarthritis syndrome, (b) bacteraemia, (c) meningitis, (d) osteomyelitis or (e) endocarditis; ii) gonococcal infection at other sites; iii) persistence of *N. gonorrhoeae* infection despite treatment (due to *in vivo* development of AMR or unknown host factor)s; iv) re-infection with the *N. gonorrhoeae* challenge strain or alternative *N. gonorrhoeae* strain during the study period; and v), adverse reactions to antimicrobial therapy, constituents in the inoculation media or inoculation procedure. Potential risks have been carefully considered, with risk mitigation strategies implemented to minimise risks to participants and the broader community, particularly to close contacts and clinical trial staff ^17^. Oversight of conduct of the clinical trial and decisions during the study will be undertaken by the Medical Monitor and SMC, comprising medically-qualified personnel and supported by specific subject matter experts in STI care and statistical analysis. The SMC will review all SAEs during the trial.

### Regulation, governance, ethics and dissemination

Infectious agents used in CHIM are not considered therapeutic goods requiring regulation by the Therapeutic Goods Administration in Australia. However, this trial has been designed to meet the high clinical and manufacturing standards expected of a contemporary CHIM. The challenge strain used in this study has been thoroughly characterized with dose manufacture processes subject to stringent quality control and release testing criteria. The procedures outlined in this provisional study protocol align with the international consensus criteria for gonorrhoea CHIM studies proposed by international experts at the inaugural Gonococcal Challenge Network Meeting in Oxford in March, 2025, that was funded by the Academy of Medical Sciences. It included consultation with community and independent experts and review by regulatory bodies including the United States Food and Drug Association (FDA) through feedback from a Pre-Investigational New Drug meeting. Publication of this provisional protocol is intended to facilitate transparent scientific peer-review prior to study commencement.

All dose analysis data and SAEs will be reviewed by the SMC, chaired by an independent clinician-scientist with appropriate experience. All dose-escalation and de-escalation recommendations will be made by the SMC. These recommendations will be reviewed by the TSC, who will approve all dose-escalation and de-escalation decisions. A broader Clinical Trial Reference Committee including community representatives, independent public health/sexual health clinicians and a bioethicist will provide advice prior to the trial to optimise acceptability of the study.

The study protocol accords with the standards outlined in the SPIRIT 2025 Statement ^70^ and the Guideline for Good Clinical Practice ^71^. It also addresses the 10 key criteria recommended by the World Health Organization (WHO) for assessment of the ethical appropriateness of a CHIM ^25^ and proposed reporting standards for CHIMs ^72^. This study protocol will be reviewed and approved by an authorised Human Research Ethics Committee (HREC), and be registered on the Australian New Zealand Clinical Trials Register (ANZCTR). Indemnity insurance for this study will be covered by a comprehensive institutional policy. Results of this study will be published in peer-reviewed journals and presented at scientific conferences to ensure dissemination of the findings to the scientific community.

## Discussion

The WHO Global Health Sector Strategy on STIs has set an ambitious target of reducing worldwide *N. gonorrhoeae* incidence by 90% by 2030 ^73^. In the absence of an effective vaccine, it is unlikely this target will be met. This oropharyngeal gonorrhoea CHIM is designed to significantly advance development of prevention and treatment strategies - including vaccines and anatomical site-specific treatment protocols - by providing a platform for early-stage evaluation and selection of promising candidates for future clinical trials. This model will simultaneously generate new insights into early oropharyngeal *N. gonorrhoeae* infection, improving our understanding of pathogenesis.

As a first-in-human CHIM of *N. gonorrhoeae* at the oropharyngeal site, with a novel challenge strain, this study design prioritises safety and acceptability, and will recruit individuals from the MSM population who are at increased risk of *N. gonorrhoeae* infection. Safety has been enhanced through rational challenge strain selection ^27^, modernization of the dose manufacture process, careful participant selection, and implementation of risk mitigation strategies to minimise adverse outcomes and the risk of transmission to close contacts and the community ^17^. The study has been informed by qualitative research and community consultation, which explored key ethical and acceptability concerns raised by both experts and potential participants, and informed study design, advertising materials, recruitment strategies, and governance ^26^.

This study protocol incorporates the outcomes of several parallel projects with the ambitious aim of modernising *N. gonorrhoeae* CHIM design. These include i) selection and characterization of a contemporary *N. gonorrhoeae* challenge strain, optimized for safety, generalizability and stability in modern cell bank manufacture ; ii) development of a *N. gonorrhoeae* cell bank manufacture protocol to enable dosing at bedside following thawing of a single-dose vial on the day of inoculation, simplifying the pre-inoculation dose preparation process, and enabling formal release testing and dose determination prior to participant inoculation; iii) use of a CRM for dose escalation, de-escalation and statistical analysis, improving the accuracy and efficiency of identifying the target challenge dose in this dose-finding study; and iv), incorporation of meaningful community consultation into study design and recruitment strategies; a key element of optimizing acceptability and cultural safety of a CHIM with a potentially stigmatising sexually-transmitted pathogen. Each element has been intentionally included to ensure alignment with international best practices for safety and scientific rigour in CHIM research.

There are inherent limitations to using gonorrhoea CHIMs for early-stage evaluation of prevention and treatment strategies. *N. gonorrhoeae* is characterized by antigenic variation and phase variability, meaning findings from a single-strain CHIM may not be generalizable to all circulating strains. To address this, we selected a challenge strain with MLST and NG-MAST sequence types representing diverse geographic regions across at least three continents between 2015 and 2021, improving generalisability ^27^. This CHIM, which uses a different strain and anatomical site from the only other active gonorrhoea CHIM (the University of North Carolina urethritis model using *N. gonorrhoeae* FA1090), can complement existing models and provide broader data to inform early-phase testing of treatment and prevention strategies before proceeding to larger, more costly field studies.

Another potential limitation relates to the generalisability of the findings arising from the oropharyngeal site to urogenital sites of infection that cause greatest morbidity. However, there are several reasons to believe the oropharynx is a key site to test novel gonorrhoea treatment and prevention strategies. Firstly, it likely plays a significant role in transmission of gonorrhoea in the community ^9^, including bridging transmission of *N. gonorrhoeae* infection from MSM to women ^74^, who are at highest risk of long-term morbidity impacts of *N. gonorrhoeae* infection ^75^. Secondly, it is a high-risk site for the development of AMR due to prolonged colonization ^13^ and horizontal transfer of AMR from commensal oropharyngeal microorganisms ^12^. Thirdly, for any prevention strategy to have long-term public health impacts, it is critical that is has efficacy at extragenital as well as genital sites. Modelling studies have estimated that a vaccine without efficacy at the oropharynx is unlikely to have a significant impact on overall population rates of *N. gonorrhoeae*, and that prevalence may actually increase if a vaccine prevents symptoms but not infection or transmission ^24^. Finally, population groups who bear the highest burden of morbidity from *N. gonorrhoeae* infection have been purposively excluded from this study, including individuals assigned female at birth, individuals who have sex with people assigned female at birth, and PLHIV. The impacts of gonorrhoea on people with a female reproductive system can be profound, and due to current limitations in understanding of *N. gonorrhoeae* transmission (including the role of autoinoculation) and female urogenital gonorrhoea pathogenesis, this group has been excluded from participation in this model for safety and ethical reasons ^17^. PLHIV have also been excluded from this study due to increased reports of disseminated gonococcal infection among PLHIV ^40, 50, 51^. Insufficient data are available regarding the differential risk of disseminated gonococcal infection among PLHIV with well-controlled HIV infection to enable risk stratification at this time. As has been planned for the pneumococcal CHIM ^76^, it is feasible that people with well-controlled HIV could be considered for inclusion in future studies if evidence suggested that such an approach would be safe and likely to yield important results for this population.

This protocol describes a first-in-human, dose-finding oropharyngeal gonorrhoea CHIM using *N. gonorrhoeae* strain AUSMDU00053933. Establishing a safe and reproducible dose required to achieve oropharyngeal infection will support the use of this model in translational research of novel treatment and prevention strategies. It will also generate novel insights into early infection dynamics and host immune responses at the oropharyngeal site. Ultimately, we hope this CHIM may contribute meaningfully to reducing the global health burden of *N. gonorrhoeae*.

## Data Availability

Figshare: CURE-NG Continual Reassessment Model Design (Extended Data); doi.org/10.26188/30285286 (Price DJ, Williams E, McCarthy JS, 2025). CURE-NG Participant Questionnaire; doi.org/10.26188/30285433 (Williams E, Hocking JS, McCarthy JS, 2025). CURE-NG Provisional Protocol SPIRIT 2025 Checklist; doi.org/10.26188/30413080 (Williams E) This project contains the following underlying data: CURE-NG Continual Reassessment Model Design (Extended Data).pdf CURE-NG Participant Questionnaire.pdf CURE-NG Provisional Protocol SPIRIT 2025 Checklist.pdf Data are available under the terms of the Creative Commons Attribution 4.0 International license (CC-BY 4.0)
