# Supplementary material for: An oropharyngeal gonorrhoea controlled human infection model: a provisional protocol using a novel Neisseria gonorrhoeae challenge strain
Source: Wellcome Open Res. 2025 Nov 19;10:636. [Version 1] doi: 10.12688/wellcomeopenres.25103.1 (PMC12902672; doi:10.12688/wellcomeopenres.25103.1)
Supplement: Peer review history

# Reviewer report: Reviewer response for version 1

**DOI:** 10.21956/wellcomeopenres.27675.r142325

**Published:** 2026-02-12

**Authors:** Victoria Miari

**Affiliations:** 1 London School of Hygiene & Tropical Medicine Faculty of Infectious and Tropical Diseases, London, UK

**Summary of the report: **Controlled challenge studies for *N. gonorrhoeae* pharyngeal infection are currently lacking; this protocol fills a much needed gap that will enable further research into how *N. gonorrhoeae* establishes pharyngeal infection and other contexts that the authors identified. The research protocol provided seems mostly thorough and robust, but I have some comments.

**Strengths: **The authors have gone in a great deal of effort in involving 'lay' persons in the design of the research which should be commended. There also seems to be plans to continue the relationship throughout the duration of the study. Further, the authors seems to have thought about key definitions in detail, for example what they consider 'infection', considering 

 1. I presume that since this is a small study assessing primarily safety and tolerability, there will be a larger subsequent study. I am interested in whether the authors have considered monitoring drop out rates - especially as this is a 90 day follow up period - and implementing a questionnaire to understand why participants may be dropping out? This data will also be useful in calculating sample sizes in future studies. This is something perhaps that the 'lay' collaborators may be able to lead on. 

 2. The authors have excluded women (or AFAB) - a patient group often excluded from research - based on the risk of auto inoculation and fertility risks. Is there any evidence that auto inoculation actually occurs from the pharynx to urogenital sites and if so, how often? The authors have proposed stringent infection control measures in MSM that presumable can be applied to any group.

 3. I am wondering if there are any plans to perform metagenomic studies or even better isolate any commensal *Neisseria* species from participant pharyngeal swabs pre Ng inoculation? There is some evidence that *N. lactamica* may provide protection against *N. meningitidis* and some in vitro studies showing some commensal *Neisseria* species can kill *N. gonorrhoeae*. Having this data may be informative for participants who do not become 'infected'. I appreciate that the sample size in this study will be too small to extrapolate anything concrete but may provide a basis for a larger study.

 4. Regarding the estimated inoculum, the authors state that the dacron swab deposits 10-fold lower cfu that the initial suspension. They have referenced a study that states this; I am wondering if they have validated this in-house? And can the authors explain why a swab inoculation method was chosen instead of a sprayed inoculum?

 5. The participants will be receiving treatment during the study - after 5 days if asymptomatic and before that if symptomatic. Have the authors considered performing molecular typing on the isolate to ensure the strain they are treating is in fact the strain inoculated and not one acquired through oral sex? I am aware that participants will be coached on proper conduct for the duration of the study but they are human after all. Having this proof key, considering one of the outcome measure is calculating colonisation rate (ID _60_ - ID _80_). Molecular typing should be possible even without a gonococcal isolate.

 6. How will the results of this study feed into subsequent larger research studies? I am particularly interested in the collected serum and salivary samples for the immunological response work. Not a lot of information is given on that aspect of the protocol. Can the authors specify the exact non-microbiological testing that will be performed on these samples?

**Stats: **I am not a stats expert so will not be able to comment on the statistical testing proposed.

Is the study design appropriate for the research question?

Partly

Is the rationale for, and objectives of, the study clearly described?

Yes

Are sufficient details of the methods provided to allow replication by others?

Partly

Are the datasets clearly presented in a useable and accessible format?

Not applicable

Reviewer Expertise:

I am a clinical academic with expertise in microbiological diagnostics/antimicrobial susceptibility testing and have developed a niche research interest in pharyngeal gonorrhoea and HGT with commensal Neisseria species.

I confirm that I have read this submission and believe that I have an appropriate level of expertise to confirm that it is of an acceptable scientific standard, however I have significant reservations, as outlined above.

# Reviewer report: Reviewer response for version 1

**DOI:** 10.21956/wellcomeopenres.27675.r142984

**Published:** 2026-01-21

**Authors:** Leshan Xiu

**Affiliations:** 1 Shanghai Jiao Tong University School of Medicine, Shanghai, Shanghai, China

The global threat of gonorrhea and its antimicrobial resistance continues to pose significant public health challenges, and currently, no effective vaccine exists. This study describes a protocol for an oropharyngeal gonorrhea controlled human infection model, designed with a strong focus on maximizing participant safety. The primary aim is to develop this model as a platform to accelerate the development of prevention and treatment strategies. The study design incorporates critical considerations for safety and tolerability, along with the determination of the minimum infectious dose of the isolate. The protocol also includes early evaluation of clinical, immunological, microbiological, and pharmacometric responses, which are pivotal for advancing future prevention and treatment strategies, including vaccine development and treatments targeting specific anatomical sites. The data generated from this study could serve as a valuable foundation for future clinical trials and offer a promising candidate model. Furthermore, this model provides new insights into oropharyngeal gonococcal infections, enhancing our understanding of the pathogenesis of the disease. However, there are some limitations to consider. The recruitment of participants has been limited to men who have sex with men (MSM). While the authors have provided reasons for this choice, the broader applicability of the model should be carefully considered, especially in the context of different populations. Additionally, the relatively small sample size of recruited participants could be a limitation. If feasible, increasing the sample size in future studies would help validate the model's robustness and generalizability.

Is the study design appropriate for the research question?

Yes

Is the rationale for, and objectives of, the study clearly described?

Yes

Are sufficient details of the methods provided to allow replication by others?

Yes

Are the datasets clearly presented in a useable and accessible format?

Yes

Reviewer Expertise:

Infectious diseases and antimicrobial resistance in Neisseria gonorrhoeae.

I confirm that I have read this submission and believe that I have an appropriate level of expertise to confirm that it is of an acceptable scientific standard.

# Reviewer report: Reviewer response for version 1

**DOI:** 10.21956/wellcomeopenres.27675.r142982

**Published:** 2026-01-08

**Authors:** Lewis C. E. Mason

**Affiliations:** 1 Department of Applied and Human Sciences, Kingston University London, London, UK, UK

**Brief introductory note**

 Firstly, I thank Wellcome Open Research for their invitation to peer review this study protocol. To be entirely transparent from the beginning, I am not medically trained, nor do I have expertise in the legal/regulatory space regarding human infection challenge studies. I have reviewed this *Neisseria gonorrhoeae *( *N. gonorrhoeae*) oropharyngeal human infection study protocol mainly based upon its scientific, epidemiological, and public health qualities. It is reassuring to see that a plethora of parties of interest have been involved in this study protocol design: community-based organisation representatives; subject matter experts; public health physicians; industry representatives; and individuals who would be eligible to participate in such a study. I defer the peer review of matters concerning bedside management, clinical decision-making, bioethics, community acceptability, and any other areas outside of my scientific expertise, to those who are authoritative sources of valid peer review in these areas.

**Overview**

*N. gonorrhoeae *is a bacterial causative agent of gonorrhoea, a sexually transmissible infection (STI) which, in higher-income nations, disproportionately infects gay, bi, and other men who have sex with men (GBMSM). ^1^ The authors describe their stipulated study protocol for an oropharyngeal controlled human infection study using a genomically-informed and epidemiologically-relevant *N. gonorrhoeae *strain. The rationale for, and objectives of, the study have been clearly described by the authors. The overall study design is generally appropriate, alongside the detailed descriptions of methodologies for reproducibility purposes. I have some comments and suggestions, which I have outlined below, regarding the study design and reproducibility of methods.

**Review of the inoculation methodology study design**

 The authors state that the starting dose is ‘ *10 ^5^ ± 0.5 log _10_ CFU/ml’*, and that this is referring to the concentration of the dose in the vial rather than the estimated dose delivered. While the authors have cited that Dacron swabs typically release a 10-fold lower concentration of bacteria than the dose in the starting vial (therefore, here, the dose given in this study would be typically around 10 ^4 ^CFU/ml), there are steps that could be employed within the protocol itself to make the actual dose inoculated more likely to be consistent.

 The authors note that ‘ *the vial will be prepared by performing gentle inversion ten times to mix the inoculum and the sterile swab will be dipped in the vial for ten seconds’ *and that ‘… *the operator will apply the inoculum by rolling the swab back and forth over the tonsillar arches and posterior oropharynx.’ *To make this application procedure more reproducibly robust, the authors could consider incorporating rigid operator training regarding many factors: the thawing procedure and thawing time-keeping of the vial; a robust methodology for the homogenisation of the contents of the vial (though ten gentle inversions may be sufficient if there is no excessive clumping or settling present); pressure of the swab on the relevant anatomical areas; the number of passes with the swab; what constitutes as a ‘ *pass*’; the surface area needed to be touched by the swab; the time of contact between the swab and the participant; the exact anatomical placement of the swab, and what exactly constitutes as adequately ‘ *rolling the swab back and forth*’; and finally perhaps also highlighting the need to record these swabbing event details for each participant.

 If feasible, a record of the mass of the Dacron swab before and after dipping into the vial could be taken to investigate variability among operators (if there are indeed multiple operators performing the inoculations) and/or variability among batches of swabs/vials following uptake of liquid into/onto the swab.

 I believe it to be fundamentally essential for this study to have a standardised inoculation checklist, a comprehensive training and competency plan for the operator(s) inoculating the participants, and a clear outline of the level of detail required to be recorded by the operator(s) of events before, during, and following the inoculation process (those being the numbers that could be collected at each stage outlined above, such as the number of passes with the swab, contact time, etc.) and any instances of the gag reflex. The authors have indicated that an ‘ *exaggerated*’ gag reflex is an exclusionary criterion, however all gag reflexes (and indeed even coughs and sneezes) – even those not considered to be ‘ *exaggerated*’ – should at least be recorded, descriptively at a minimum, as this may impact the deposition of the *N. gonorrhoeae *in the anatomical region and perhaps account for variability in the minimum inoculum dose of *N. gonorrhoeae *among the participants.

 One cofounding factor in the minimum inoculation dose of the *N. gonorrhoeae *in the participants may be variability in the amount of saliva produced among the participants, and this could be systematically investigated (via a salivary flow rate test) at the inoculation stage and be pre-emptively recorded for ease of interpretation in the future if unexplainable variations do indeed occur.

 The authors may also wish to also consider, following the development of a robust swab-liquid uptake and inoculation protocol, quantitatively deducing the likely dose given to each participant by following the protocol at the bench and inoculating onto agar to then determine the colony forming unit (CFU) count and evaluate the reproducibility of the liquid-uptake and inoculation techniques employed among swab/vial batches and technique of the operator(s).

**Review of the eligibility and exclusion criteria study design**

 The authors note that as part of their eligibility criteria, ' *drug/alcohol misuse*' is listed for exclusion, specifically ' *...illicit drug use, or any prior intravenous usage of an illicit substance*', as per Table 3 in the study protocol. Also, that during the study ' *...participants must not use illicit drugs or alcohol' *(Table 4). I think that it may be worth the authors clearly outlining what are considered 'illicit' drugs/substances for the purposes of this study protocol, given the behaviourally disinhibiting effects of some substances that may not be technically 'illicit' (if one interprets 'illicit' to simply mean 'illegal') in Melbourne, Australia. For example, the ‘chemsex’ party-drug group of alkyl nitrites (colloquially known as 'poppers') which are typically legally sold as commercial products such as leather cleaners, Video Home System (VHS) head cleaners, solvents, air fresheners, among many others.

 While specific sexual activities with others are clearly prohibited while being a participant of this study (Table 4), alkyl nitrites are not solely used for their analgesic vasodilatory muscle-relaxing properties during ‘chemsex’, and may also be taken in social gathering settings simply for their euphoric and ‘warming’ effects, which could inadvertently lead to disinhibition of sexual behaviours and therefore potential non-compliance with the study participation guidelines. In addition, as alkyl nitrites are taken via inhalation through the nose and/or mouth (leading to the vapours being exposed to the oropharyngeal region and nasopharynx), this could have unintended consequences for the localised microbiota and any *N. gonorrhoeae *inoculated in the region.

 Indeed, it may be that historical use of poppers should also be considered for eligibility purposes due to the caustic and irritating nature of the substance(s) which may have caused persistent anatomical changes in the oro/nasopharyngeal region (lesions, burns, irritation, even potentially a changed microbiota) which could confound the results of this human infection challenge study. I note that the authors are employing urine-based drug-screening in their study, which would typically be unable to identify the presence of alkyl nitrites due to their rapid metabolisation and excretion through urine very soon after inhalation.

 I note that the authors have stated that exclusion criteria also include ‘ *history of any clinically important cardiac, endocrinologic, haematologic, hepatic, immunologic, metabolic, urologic, pulmonary, neurologic, dermatologic, psychiatric, renal or other major disease, as determined by the Investigator*’. It may be worth the authors explicitly outlining, at this early stage, a comprehensive list of specific associated conditions which would undoubtedly come under these exclusionary criteria due to their anatomical and immunological relevance. The authors have, understandably, explicitly excluded those with a history of tonsillectomy and adenoidectomy, though the authors may also wish to consider those who have had uvulopalatopharyngoplasty (UPPP), uvulectomy, palatoplasty, among many other procedures, particularly as participants may not be immediately aware of having had these surgical procedures if they were undertaken at a young age and may not be readily available in their medical history. A record of anatomical variations in the participants, resulting in the need to deviate from the prescribed inoculation protocol by the operator(s), should be recorded.

 The authors have stated that ‘ *use of vitamin supplements taken at standard doses is allowed*’, in the consideration of exclusionary criteria (Table 3). The authors may wish to reconsider their allowance of the use of vitamin supplements (and also mineral supplements) during the study period, even at ‘ *standard*’ dosage (and if the authors elect to continue to allow supplement use, ‘ *standard doses’* should be explicitly defined, and a record taken from each participant for which supplements, at what dose(s), and whether or not the participant has been determined to be deficient in the associated supplement previously). The reasoning for this is that some vitamin/mineral supplements may impact the gastrointestinal tract microbiome (including the oropharyngeal cavity), particularly in the case of iron supplementation when taken with no known deficiency. ^2^

 Also, *N. gonorrhoeae *requires iron to proliferate (as do most bacteria) which may inadvertently impact the minimum inoculum dose of the challenge study whereby those who take iron supplements may be inadvertently more susceptible to colonisation by *N. gonorrhoeae*, or have a changed salivary microbiome which may influence the results of this study. Likewise, iron deficiency may also impact the oral microbiome which could inadvertently affect the results of the study.

 Another exclusionary criterion stipulated by the authors is being a ‘ *current active smoker defined as having smoked a cigarette or cigar in the four weeks prior to challenge*’. The authors may wish to consider also explicitly extending this to include active ‘vapers’ (e-cigarette users) who have ‘vaped’ in the four weeks prior to the challenge. It has been previously established that e-cigarette use alters the oral/salivary microbiome. ^3^

 The authors may wish to edit ‘ *participants must not engage in sexual activity including contact with the mouth (including deep kissing and saliva use in sexual activity), or contact with the penis, urine, semen or anorectal region with any other individual*’ in Table 4 to also include contact with the vagina. While the participant requirement is indeed ‘ *male volunteers who exclusively have sex with people assigned male (assigned at birth)*’, there may be participants who have sexual partner(s) that were assigned male at birth who now have a vagina via genital surgery. It may also be prudent to expand the guideline to be ‘ *must not engage in* *any and all sexualised contact and activities, including but not limited to…*’, as the participants may theoretically (though unlikely) be able to transmit their inoculated *N. gonorrhoeae* to sexual partner(s) through the handling of sex toys (not necessarily ‘ *sharing*’ the use of, which the authors have already prohibited in the participant guidelines in Table 4), as the risk of even handling sex toys to use on their partner may not be immediately apparent to the participant(s) to be contrary to the participant guidelines (due to residual self-derived saliva/semen/rectal mucus left on their hands, etc.). The handling/sharing of non-sexual fomites (towels, etc., beyond just cutlery and drinking vessels) may also be worth indicating as contrary to the guidelines. There are also other sexualised activities involving bodily fluids beyond saliva/semen/urine, such as faecal (‘scat’)-play, which may not be immediately apparent to be contrary to the participant guidelines, but could theoretically facilitate onward transmission of the *N. gonorrhoeae *from the oral cavity if endogenous transmission within the host occurs.

 Advising participants regarding the risk of endogenous infection spread from their orally inoculated *N. gonorrhoeae *to their own penile and anorectal region may also be prudent. Such an event may occur if a participant uses their saliva as lubricant during masturbation, oral-anal sex toy use, auto-anal-digital contact (self-‘fingering’/’fisting’), autofellatio (self-oral-penile masturbation), self-use of urethral sounding rods, among other activities, as Table 4 currently only seems to guide against sexual activities ‘ *with any other individual*’ and not necessarily oneself.

**Review of the challenge strain antimicrobial treatment study design**

 The authors note that in the scenario of treatment failure of the challenge strain with ceftriaxone (less likely, due to the initial genomic content of the strain used indicating susceptibility to a wide range of antimicrobials, confirmed via antimicrobial susceptibility testing), the *N. gonorrhoeae *cultured from the participant will undergo whole genome sequencing (WGS) and phenotypic antimicrobial susceptibility testing ‘ *to assess the cause of potential treatment failure*’. The authors may wish to require all participants to keep a record of consumption of food, drink, vitamin and mineral supplements (if the authors elect to continue to allow their use), and any non-antibiotic medications (NAMs) that they may have taken during the study period. There is an increasing awareness among the scientific community that microbial exposure to NAMs may indeed facilitate an environment of selective pressure for the evolution of antimicrobial resistance, via multiple molecular mechanisms previously outlined elsewhere, ^4,5 ^and also facilitate changes in the abundance and diversity of microbes within the gastrointestinal tract microbiome.

 As the authors have elected to have ‘ *sporadic use of non-steroidal anti-inflammatory drugs*’ (e.g. ibuprofen, aspirin, etc.) not be an exclusionary criterion for participating in the study, it is important that the participants record their use of NAMs – particularly if needed in the near future to assess potential causes of treatment failure and/or variability in the colonisation and minimum inoculum doses of *N. gonorrhoeae* among the participants. The authors may wish to consider excluding those who have used NAMs during the study, due to this phenomenon of NAM-mediated selection pressure for the evolution of antimicrobial resistance in exposed microbes, and disruption of the microbiome, which is currently being extensively investigated by the scientific community.

 I also express tentative concern regarding the decision to treat ‘ *all participants*’ with ceftriaxone, particularly in cases where *N. gonorrhoeae *infection (true colonisation) is not confirmed to have been established in the participant. While understandable from one viewpoint of participant safety (where preventing unnoticed colonising and proliferation of *N. gonorrhoeae *during the initial study period is concerned), treatment with ceftriaxone is not without its risks – also including in regards to the gut microbiome of the participant, ^6^ considering the impact on the diversity and abundance of specific microorganisms but also the potential impact on the resistome in the participant. Also, as the participants are being followed up for three months following their initial participation, there may be abundant opportunity to identify true microbiologically-confirmed *N. gonorrhoeae *colonisation and/or delayed symptomatic infection events, and hence appropriate treatment with ceftriaxone, beyond the maximum ‘wait’ period of only five days in the initial participation protocol.

 The authors should also make the participants aware of specific NAMs that may be associated with poor clinical outcomes when taken in combination with ceftriaxone, the most relevant to immediately come to mind being proton pump inhibitors (PPI), ^7^ though there may be others. I defer the comprehensive review of this to clinically trained colleagues.

 While the authors have stated that STI screening will be undertaken as part of the pre-screening for eligible participants, it is not clear whether carriage of any/all sexually transmissible pathogen(s) would also exclude an individual from participating in the study (beyond carriage of HIV and *N. gonorrhoeae, *which have been clearly outlined by the authors as exclusionary criteria). Sexually transmissible enteric pathogens, such as *Campylobacter *spp., *Shigella *spp., *Salmonella *spp., and certain *Escherichia coli *pathotypes, among many other species, ^8^ transmitted via the faecal-oral route during sexual activities involving anal-digital and anal-oral contact, are known to disproportionately infect GBMSM via this route – though are not usually routinely tested for with traditional, and even ‘enhanced’, STI screening. The potential for asymptomatic carriage of sexually transmissible enteric pathogens has also been previously established, ^9 ^alongside patterns of bystander resistance ^10^ influenced by the changing treatment regimens of other sexually transmissible infections (such as *N. gonorrhoeae*) whereby these enteric pathogens are exposed to antimicrobials during the treatment of other sexually transmissible infections (gonorrhoea, syphilis, etc.) and are becoming increasingly multidrug resistant (MDR) and extensively drug resistant (XDR) themselves likely partly due to this exposure. This phenomenon must also be considered where there have been any previous instances of antimicrobial treatment in participants, particularly for the treatment of sexually transmissible infections that may predate the three-month exclusionary criteria for gonorrhoea in this study.

 While the *N. gonorrhoeae *strain used in this challenge study is initially genomically susceptible to a plethora of antimicrobials, the current resistome of the participant must be considered due to the potential for treatment failure of the challenge strain via horizontal gene transfer (HGT) of antimicrobial resistant determinants to the challenge strain from the participant microbiome. I defer, to medically trained colleagues, the determination of the clinical assessment of balancing the risk between treatment/non-treatment with ceftriaxone in participants who do not have seem to have become infected (truly colonised) with the *N. gonorrhoeae *challenge strain – though it would seem essential to inform participants of the potential impact on their gastrointestinal microbiome (including resistome) and the implications of this for themselves and their current/future sexual partner(s).

 The authors may wish to consider including pre-screening the oral microbiomes of potential participants for antimicrobial resistance determinants (genes, plasmids, etc.) through 16S rRNA microbiome sequencing of the saliva and other oropharyngeal regions.  This could inform exclusionary criteria based upon the presence of relevant antimicrobial resistance determinants (those likely to cause treatment failure with ceftriaxone) that could likely be transferred to the *N. gonorrhoeae *challenge strain from commensal oral bacteria. This could be followed by oral/faecal/urine 16S rRNA microbiome sequencing subsequent to treatment with ceftriaxone during the study, to evaluate the potential resistome burden induced by its use in this study.

 The authors mention that if a participant makes the study staff aware of ‘ *infection control breaches’*  during the study period, that the study staff will facilitate contract tracing. The authors should make explicitly clear what events would constitute as an ‘ *infection control breach’* worthy of this response – does this include any and all behaviours contrary to the participant guidelines in Table 4, including the sharing of cutlery/drinking vessels? How would this be communicated in a way that remains anonymous, while sufficiently informing the traced individual of the identified behaviour (since the infection risk between sexual activity and sharing of fomites may not necessarily be equal, and is the traced individual required to be informed of the route of transmission)?

**Review of the study design of microbiologically-confirmed *N. gonorrhoeae* being via nucleic acid amplification testing (NAAT)**

 The authors have stated that the case definition of microbiologically-confirmed *N. gonorrhoeae *for this study protocol, via nucleic acid amplification testing (NAAT), is the ‘ *detection of N. gonorrhoeae by NAAT with two different N. gonorrhoeae genomic targets from a combined oropharyngeal swab collected from the posterior oropharynx, palatine tonsils and saliva.*’ This approach requires great caution, and is the major methodological concern with the study protocol as it currently stands, as the detection of *N. gonorrhoeae *DNA in the oropharyngeal cavity >48 hours post-inoculation may not necessarily equate to colonisation of the oropharyngeal region by *N. gonorrhoeae*, and could simply be remnant *N. gonorrhoeae *DNA in the oral cavity following the inoculation procedure – particularly if the inoculum dose remains inconspicuously trapped in the oropharyngeal tonsillar crypts. This is further confounded by the decision to use a combined swab of multiple sites and the saliva of the participant, which could further result in residual *N. gonorrhoeae *DNA – not associated with actual colonisation – to be inadvertently recorded as being microbiological confirmation of infection (particularly as saliva is being included).

 I note that in the text, the authors state that ‘ *the case definition… can be established via microbiological criteria alone, or combined clinical and microbiological criteria*.’, contrary to Figure 2 in the study protocol which appears to illustrate that both microbiologically-confirmed *N. gonorrhoea *infection and indicative symptoms or examination signs are findings that are needed at the same time in order to define a case occurrence. The authors may wish to consider making this clearer in the protocol.

 Due to the tendency of *N. gonorrhoeae *infection in the oropharyngeal cavity to be asymptomatic, it is an understandable approach by the authors to not solely rely on symptoms for confirmation of infection – though it would seem prudent to increase the NAAT-defined infection case definition as being where two consecutive days of positive-NAAT detection of *N. gonorrhoeae *>48 hours post-inoculation, and/or requiring both microbiological and symptom/examination based indications to be present for a case definition as it seems to be presented in Figure 2 of the study protocol. Though, I defer this to the authors’ discretion for how to better approach case definition.

**Conclusion**

 To conclude, this *N. gonorrhoeae *oropharyngeal human infection study protocol has clearly been designed carefully with the involvement of a range of parties of interest and their expertise. I also look forward to reading the forthcoming publication regarding the specifics of the community engagement undertaken in the design of this study protocol, as this is undoubtedly essential for determining the community acceptability of this study. The authors have clearly stated the rationale and objectives of their study, and, with further considerations and clarifications regarding the study design and efforts to improve reproducibility, this study would appear to be highly impactful for the scientific and public health epidemiological understanding of *N. gonorrhoeae *transmission among populations at a higher risk of transmission in a higher-income nation, and the development of community-acceptable interventions in reducing the burden of *N. gonorrhoeae *infection through vaccination and other holistic means.

Is the study design appropriate for the research question?

Partly

Is the rationale for, and objectives of, the study clearly described?

Yes

Are sufficient details of the methods provided to allow replication by others?

Partly

Are the datasets clearly presented in a useable and accessible format?

Yes

Reviewer Expertise:

My areas of expertise are predominantly microbial genomic epidemiology, antimicrobial resistance, microbial bioinformatics, phylogenetic analyses, and general bacteriology. I have worked extensively to investigate the evolution and spread of multidrug resistant (MDR) and extensively drug-resistant (XDR), sexually transmissible, Shigella species. I have experience undertaking patient and public involvement and engagement (PPIE) to evaluate community acceptability of my research, which focusses on sexually transmissible enteric infections among men who have sex with men (MSM).

I confirm that I have read this submission and believe that I have an appropriate level of expertise to confirm that it is of an acceptable scientific standard, however I have significant reservations, as outlined above.

## References

[1] : ECDC publishes the annual epidemiological report 2012. *Eurosurveillance* .2013;18(10) : 10.2807/ese.18.10.20418-en 10.2807/ese.18.10.20418-en 23515064

[2] : Iron Supplementation at the Crossroads of Nutrition and Gut Microbiota: The State of the Art. *Nutrients* .2022;14(9) : 10.3390/nu14091926 10.3390/nu14091926 PMC910203935565894

[3] Pushalkar S, Paul B, Li Q, Yang J, et al.: Electronic Cigarette Aerosol Modulates the Oral Microbiome and Increases Risk of Infection. iScience. 2020; 23 (3).10.1016/j.isci.2020.100884PMC711356432105635

[4] : Extensive impact of non-antibiotic drugs on human gut bacteria. *Nature* .2018;555(7698) : 10.1038/nature25979 623-628 10.1038/nature25979 PMC610842029555994

[5] : Non-antibiotics disrupt colonization resistance against enteropathogens. *Nature* .2025;644(8076) : 10.1038/s41586-025-09217-2 497-505 10.1038/s41586-025-09217-2 PMC1235017140670795

[6] : Antibiotic-related dysbiosis of gut microbiota in healthy volunteers: a comparative study of ceftazidime-avibactam, piperacillin-tazobactam, and ceftriaxone. *European Journal of Clinical Microbiology & Infectious Diseases* .2025;44(9) : 10.1007/s10096-025-05184-8 2151-2158 10.1007/s10096-025-05184-8 40504338

[7] Mitsuboshi S, Imai S, Kizaki H, Hori S: Concomitant use of lansoprazole and ceftriaxone is associated with an increased risk of ventricular arrhythmias and cardiac arrest in a large Japanese hospital database. Journal of Infection. 2024; 89 (2). Publisher Full Text.10.1016/j.jinf.2024.10620238897240

[8] : British Association of Sexual Health and HIV (BASHH) United Kingdom national guideline for the management of sexually transmitted enteric infections 2023. *International Journal of STD & AIDS* .2023;34(9) : 10.1177/09564624231168217 588-602 10.1177/09564624231168217 37247427

[9] : Estimating the proportion of bystander selection for antibiotic resistance among potentially pathogenic bacterial flora. *Proceedings of the National Academy of Sciences* .2018;115(51) : 10.1073/pnas.1810840115 10.1073/pnas.1810840115 PMC630494230559213

[10] Tsai C, Lee N, Chen P, Chen S, et al.: Asymptomatic enteric pathogen carriage and its association with proton pump inhibitors use in men who have sex with men in Taiwan, 2019-2022. International Journal of Infectious Diseases. 2024; 149. Publisher Full Text.10.1016/j.ijid.2024.10727339442749
